# Molecular taxonomy and evolutionary relationships in the Oswaldoi-Konderi complex (Anophelinae: *Anopheles*: *Nyssorhynchus*) from the Brazilian Amazon region

**DOI:** 10.1371/journal.pone.0193591

**Published:** 2018-03-05

**Authors:** José Ferreira Saraiva, Raimundo Nonato Picanço Souto, Vera Margarete Scarpassa

**Affiliations:** 1 Laboratório de Genética de Populações e Evolução de Mosquitos Vetores de Malária e Dengue, Coordenação de Biodiversidade, Instituto Nacional de Pesquisas da Amazônia, Manaus, Amazonas, Brazil; 2 Laboratório de Arthropoda, Departamento de Ciências Biológicas e da Saúde, Universidade Federal do Amapá, Macapá, Amapá, Brazil; University of Otago, NEW ZEALAND

## Abstract

Recent studies have shown that *Anopheles oswaldoi* sensu lato comprises a cryptic species complex in South America. *Anopheles konderi*, which was previously raised to synonymy with *An*. *oswaldoi*, has also been suggested to form a species complex. *An*. *oswaldoi* has been incriminated as a malaria vector in some areas of the Brazilian Amazon, Colombia, Peru and Venezuela, but was not recognized as a vector in the remaining regions in its geographic distribution. The role of *An*. *konderi* as a malaria vector is unknown or has been misattributed to *An*. *oswaldoi*. The focus of this study was molecular identification to infer the evolutionary relationships and preliminarily delimit the geographic distribution of the members of these complexes in the Brazilian Amazon region. The specimens were sampled from 18 localities belonging to five states in the Brazilian Amazon and sequenced for two molecular markers: the DNA barcode region (*COI* gene of mitochondrial DNA) and Internal Transcribed Spacer 2 (ITS2 ribosomal DNA). *COI* (83 sequences) and ITS2 (27 sequences) datasets generated 43 and 10 haplotypes, respectively. Haplotype networks and phylogenetic analyses generated with the barcode region (*COI* gene) recovered five groups corresponding to *An*. *oswaldoi* s.s., *An*. *oswaldoi* B, *An*. *oswaldoi* A, *An*. *konderi* and *An*. sp. nr. *konderi*; all pairwise genetic distances were greater than 3%. The group represented by *An*. *oswaldoi* A exhibited three strongly supported lineages. The molecular dating indicated that the diversification process in these complexes started approximately 2.8 Mya, in the Pliocene. These findings confirm five very closely related species and present new records for these species in the Brazilian Amazon region. The paraphyly observed for the *An*. *oswaldoi* complex suggests that *An*. *oswaldoi* and *An*. *konderi* complexes may comprise a unique species complex named Oswaldoi-Konderi. *Anopheles oswaldoi* B may be a potential malaria vector in the extreme north of the Brazilian Amazon, whereas evidence of sympatry for the remaining species in other parts of the Brazilian Amazon (Acre, Amazonas, Pará and Rondônia) precluded identification of probable vectors in those areas.

## Introduction

Neotropical region is among the most diverse in the world, particularly in north South America. Consequently, it has been a target of research focused on elucidating the origin of biological diversity and speciation models [1]. This biodiversity extends to Anophelinae mosquitoes [2]. In the most species studied, the findings suggest that the diversification process started in the Pliocene and Pleistocene epochs [3,4]. In Neotropical region, morphological analysis along with the use of multiple molecular markers have contributed to the recognition of new cryptic species complexes in anophelines, especially in the *Nyssorhynchus* subgenus, such as *An*. *albitarsis* [5], *An*. *benarrochi* [6,7], *An*. *nuneztovari* [4,8–10], *An*. *triannulatus* [11,12], *An*. *oswaldoi* [13–16], *An*. *konderi* [7,14,16], and the *An*. *strodei* subgroup [17], and in the *Kertezia* subgenus, such as *An*. *cruzii* [18,19]. Recently, a study demonstrated that Brazilian populations of the main malaria vector, *An*. *darlingi*, may comprise a species complex [20].

*Anopheles* (*Nyssorhynchus*) *oswaldoi* s.l. is distributed throughout South America east of the Andes, including in Bolivia, Brazil, Colombia, Ecuador, Guyana, French Guyana, Suriname, Paraguay, Peru, Venezuela, and the northern provinces of Argentina. In Central America, it extends to Costa Rica, Panama, and Trinidad [21,22]. *An*. *konderi* s.l. has been reported in Brazil (Acre, Amazonas, Amapá, Pará, Rondônia, Espírito Santo, Rio de Janeiro, Paraná and Mato Grosso), Peru (Loreto) and Bolívia (Cochabamba) [23–25]. Since the resuscitation of *An*. *konderi* s.l. is a recent event [26], its geographic distribution may be broader than current records indicate.

The taxonomic problem involving *An*. *oswaldoi* s.l. has existed for a long time, since its original description. Initially, *An*. *oswaldoi* (Peryassú) was described as *Cellia oswaldoi* based on the adults captured in Vale do Rio Doce and Baixada Fluminense in the states of Espírito Santo and Rio de Janeiro, respectively, in Brazil [27], without the designation of a holotype. One year later, it was synonymized with *An*. *tarsimaculatus* (currently as *An*. *aquasalis*) [28]. Subsequently, Bonne [29] recognized two races, one exophilic and characterized by a small dark ring on the hind tarsal second segment, equivalent to *An*. *oswaldoi*, and another reported in coastal areas, equivalent to *An*. *aquasalis*. Root [30] synonymized these races based on the male genitalia. Later, *An*. *oswaldoi* was resurrected from synonymy with *An*. *tarsimaculatus* based on the morphological differences in the adults and immature stages [31], and *An*. *aquacaelestis* was described as a variety of *An*. *tarsimaculatus* in Panamá [32]. Senevet and Abonnenc [33] synonymized *An*. *aquacaelestis* with *An*. *oswaldoi*. Galvão and Lane [34] proposed three subspecies for *An*. *oswaldoi*: *An*. *oswaldoi oswaldoi*, *An*. *oswaldoi metcalfi*, and *An*. *oswaldoi noroestensis*. Galvão and Damasceno [23] described *An*. *konderi* from specimens collected in Coari, state of Amazonas, Brazil, reporting differences in the apical region of the mesosoma of the male genitalia. In the same publication, the authors considered *An*. *noroestensis* (currently as *An*. *evansae*) to be a distinct species from *An*. *oswaldoi* and *An*. *metcalfi*, whereas *An*. *metcalfi* was considered a *nomen dubium*. Lane [32] considered *An*. *konderi* and *An*. *oswaldoi* to be morphologically indistinguishable based on the egg, 4^th^ instar larvae, pupae and adult females [36,37] and synonymized these taxa. In fact, they can only be distinguished by the apical portion of the mesosoma of the male genitalia [23]. *An*. *konderi* has a rounded mesosoma that is wider than it is long, with a small lateral protuberance on both sides, whereas *An*. *oswaldoi* has an elongated mesosoma, rounded and narrow, but without lateral protuberances. Lounibos et al. [35] were unable to differentiate the morphological structure of the eggs of the two species based on scanning electron micrographs. Flores-Mendoza et al. [26], based on differences in the apical portion of the mesosoma of the male genitalia, resurrected *An*. *konderi* from synonymy with *An*. *oswaldoi*, re-describing all stages and designating a neotype for the former. These differences were supported by Motoki et al. [15], who also observed an indistinguishable ventral claspette between them. Since Peryassú [36] did not designate a type in the original description, Motoki et al. [15] re-described and designated a lectotype to fix the identity of the nominotypical of *An*. *oswaldoi* s.s.

In ecological terms, *An*. *oswaldoi* s.l. has been described as a typically exophilic and zoophilic mosquito; it has often been collected in primary tropical forest or in well-preserved forested areas [21,37–42]. However, it has been captured in open areas and has been known to bite humans in outdoor environments [39,40,42–44]. The peak biting activity of this species has been reported between 18:00 and 20:00 hours, ceasing after 21:00 hours [37,42]. In Venezuela, however, *An*. *oswaldoi* s.l. has been captured in the indoor environment and continues to bite until approximately midnight [45,46]. Klein and Lima [38] proposed that, although *An*. *konderi* is often mistaken for *An*. *oswaldoi*, *An*. *konderi* is present in human impacted or open areas and is collected throughout the year, whereas *An*. *oswaldoi* is restricted to forested areas and is only collected in large numbers from March to July.

*An*. *oswaldoi* s.l. has been reported to be naturally infected with human malaria parasite in several Brazilian states [42,47–53], Colombia [54], Peru [55], Venezuela [45,56] and French Guyana [57]. The importance of *An*. *konderi* as a malaria vector is largely unknown. Under laboratory conditions, *An*. *oswaldoi* s.l. has been shown to be susceptible to infection with *Plasmodium vivax* and *P*. *falciparum* [58], but Klein et al. [59] demonstrated a low infection rate and low numbers of sporozoites in this anopheline when compared, for example, with *An*. *triannulatus*. Also under experimental conditions, Marrelli et al. [58], using *P*. *vivax*, observed that both *An*. *oswaldoi* and *An*. *konderi* developed oocysts in the midgut, but the complete cycle of the parasite, with sporozoites reaching the salivary glands, was only observed in *An*. *oswaldoi*. This result suggests that *An*. *oswaldoi* may be more susceptible than *An*. *konderi* and may potentially transmit *P*. *vivax* under natural conditions.

The variation observed in the morphology, behavioral patterns and ecologic niches across the geographic distribution of *An*. *oswaldoi* has led many authors to suggest that it could be a cryptic species complex [11,23,38]. Therefore, population genetics studies with molecular markers could help to clarify its cryptic diversity and taxonomic status. To achieve this goal, the first molecular study with ITS2 sequences was conducted by Marrelli et al. [13]. The authors observed four groups of *An*. *oswaldoi* s.l. across Brazil, Peru and Venezuela, of which one could represent *An*. *konderi* and the remaining groups could represent *An*. *oswaldoi* s.s. and possibly a new species within *An*. *oswaldoi* s.l. A few years later, Marrelli et al. [60] observed that an *An*. *oswaldoi* specimen from Espírito Santo, Brazil, sequenced by Marrelli et al. [13] could in fact correspond to *An*. *evansae*, and other specimens of *An*. *oswaldoi* s.l. from Yurimaguas, Peru, also sequenced by Marrelli et al. [13], could correspond to *An*. *benarrochi* B [6]. In that same study, Ruiz et al. [6] molecularly identified a distinct lineage of *An*. *oswaldoi* s.l. of Putumayo in southern Colombia, which was later informally named *An*. *oswaldoi* B [61]. The sequences of this anopheline showed a high similarity with specimens from the state of Amapá in Brazil (98.76%) and Ocamo in Venezuela (99.20%) [13]. Isozyme study did not reveal differences between *An*. *oswaldoi* s.l. (possibly *An*. *oswaldoi* s.s.) and *An*. *konderi* s.l. (possibly *An*. *konderi* and *An*. sp. nr. *konderi* (= *An*. species near to *konderi*)) from the Brazilian Amazon [62]. The sequencing of the *COI* long fragments of the specimens from the four localities of the Brazilian Amazon generated four strongly supported clades that corresponded to *An*. *oswaldoi* s.s. (Acre and Amazonas), *An*. *oswaldoi* A (Pará), *An*. *konderi* (Rondônia) and *An*. sp. nr. *konderi* (Acre and Rondônia) [14]. Later, Motoki et al. [25], analyzing samples of the *An*. *konderi* complex from the states of Acre, Amapá, Rondônia and Paraná (Brazil) with ITS2 and *COI* markers, suggested three species. More recently, Ruiz-Lopez et al. [16], in a systematic molecular study, reported *An*. *oswaldoi* s.s., *An*. *oswaldoi* A and *An*. *konderi* of Sallum in Brazil; *An*. *oswaldoi* B in Colombia, Ecuador and Trinidad and Tobago; and *An*. sp. nr. *konderi* in Colombia, Ecuador and Peru. These data, combined with previous studies [7,13,14,25,63], indicated that there were three species in the *An*. *oswaldoi* complex (*An*. *oswaldoi* s.s., *An*. *oswaldoi* A, and *An*. *oswaldoi* B) and two species in the *An*. *konderi* complex (*An*. *konderi* and *An*. sp. nr. *konderi*). However, Ruiz-Lopez et al. [16] analyzed few localities (n = 3) and only seven specimens from the Brazilian Amazon, leaving a large gap in this region.

The present study analyzed the molecular taxonomy, genealogical relationships among haplotypes, phylogenetic inferences, including the diversification time, and genetic distances of the members of the *An*. *oswaldoi* and *An*. *konderi* complexes from the Brazilian Amazon using two molecular makers: the DNA barcode region of the *COI* gene from mitochondrial DNA (mtDNA) and the Internal Transcribed Spacer 2 (ITS2) of the ribosomal DNA (rDNA). The geographic distribution of the species and their probable areas of sympatry in the Brazilian Amazon were also preliminarily inferred in this study.

## Materials and methods

### Mosquito collection and morphological identification

The choice of localities was based on the sampling gaps from previous studies [13,14,16,25], probable areas of sympatry [7,14] and the records of *An*. *oswaldoi* s.l. infected with *Plasmodium* spp. in the Brazilian Amazon [41,42,49–51,53]. The specimens were collected from 18 localities distributed in five states from the Brazilian Amazon: Acre (3), Amapá (7), Amazonas (5), Rondônia (2) and Pará (1). The collections were performed between February 2013 and November 2014, except for the specimens from the states of Acre, Amazonas (Coari) and Rondônia, which were provided by the author of this study (VMS), and those from the Lábrea (Amazonas) and Serra do Cachorro (Pará) provided by Dr. Ronildo Alencar. Here, we will follow the same designation provided by Ruiz-Lopes et al. [16] for the species *An*. sp. nr. *konderi* (= *An*. species near *konderi*), which has not yet been described. **Table 1** provides the information regarding the sampled sites, states, geographic coordinates, sample sizes, and number of individuals analyzed for each marker. The coordinates were converted to Universal Transverse Mercator (UTM). The collections were authorized by the Brazilian Institute for the Environment and Renewable Natural Resources (IBAMA) and the System of Authorization and Information in Biodiversity (SISBIO), with license number 38440 awarded to VMS.

**Table 1:** Information regarding the collection sites, including the states, municipalities and locations, geographic coordinates, sample sizes and number of individuals analyzed for each molecular marker.

State	Municipality	Locality	Coordinates in UTM		N	Sample size		*An*. *oswaldoi* s.s.	*An*. *oswaldoi* A	*An*. *oswaldoi* B	*An*. *konderi*	*An*. sp. nr. *konderi*
			Latitude	Longitude		*COI*	ITS2					
Acre	Rio Branco	Senador Guiomar	-9.93266	-67.8667	5	5	2	2				3
	Sena Madureira	Sena Madureira	-9.05	-68.65	1	1	1					1
	Acrelândia	Rodovia Transacreana	-8.07663	-71.3935	2	2	1	1				1
Amazonas	Autazes	Autazes	-3.69916	-59.1318	11	11	0				11	
	Coari	Igarapé do Isidoro	-0.08333	-63.1333	5	5	3	2	3			
	Lábrea	Lábrea	-7.66452	-65.0697	12	12	0		12			
	Nova Olinda do Norte	Rodovia AM 254	-3.83694	-59.0215	1	1	1					1
	Presidente Figueiredo	Pitinga	-0.78238	-60.0604	4	4	3		4			
Amapá	Calçoene	Lourenço	2.45713	-51.2675	1	1	1		1			
	Ferreira Gomes	Paredão	0.83555	-51.2087	3	3	2			3		
	Macapá	Mata Fome	0.21266	-50.9727	4	4	4		1		3	
	Macapá	Fazenda SantaBarbara	0.29122	-50.902	2	2	1		1		1	
	Santana	Ilha de Santana	-0.08383	-51.1637	3	3	0				3	
	Serra do Navio	Pedra Preta	0.89363	-52.0116	6	4	2			4		
	Tartarugalzinho	Tartarugalzinho	1.51641	-50.9171	1	1	1			1		
Pará	Oriximiná	Serra do Cachorro	-1.002	-57.1265	14	7	4		3		4	
Rondônia	Porto Velho	Parque municipal	-8.70822	-63.9332	2	2	0				1	1
	São Miguel	São Miguel	-0.14333	-63.8166	18	15	1				15	
**Total**					**95**	**83**	**27**	**5**	**25**	**8**	**38**	**7**

UTM: Universal Transverse Mercator; N: sample size; *COI*: Cytochrome oxidase, subunit I; ITS2: Internal transcript spacer 2.

Adult female mosquitoes were captured using a light trap, white Shannon-type [64], installed at the border of the forest, between 18:00 and 22:00 hours. The captured specimens were placed in collection cups and transported alive in polystyrene boxes to the Laboratory of Population Genetics and Evolution of Vectors Mosquitoes at Instituto Nacional de Pesquisas da Amazônia (INPA) in Manaus, Brazil, where all subsequent analyses were performed. The immature stages were collected from the borders of the breeding sites, preferably with vegetation (aquatic plants), using a long-handled ladle between 7:00 and 10:00 hours. The specimens collected were transported to the laboratory, where they were reared to adulthood in the insectary, at a temperature ranging from 26°C to 28°C and relative humidity from 80% to 90%. The adults were killed in the freezer at –20°C and identified using taxonomic keys [32,65]. It was not possible to separate *An*. *oswaldoi* from *An*. *konderi* since only females were captured. The specimens were then preserved in 95% ethanol and stored in the freezer at -20°C until extraction of genomic DNA.

### Genomic DNA extraction, PCR amplification and sequencing of the *COI* and ITS2 markers

Genomic DNA was isolated from the legs of mosquito adults using the phenol-chloroform protocol [66], and the DNA pellet was resuspended in 30 μL of 1x TE buffer (10 mM Tris-Cl pH 8.0; 1 mM EDTA pH 8.0) or sterile water. A small aliquot of this DNA was stored at –20°C and then used as template for amplification by polymerase chain reaction (PCR) of the two proposed markers. The remaining aliquots of the DNA were kept frozen as the DNA-voucher at –85°C in the mosquito collection at the Laboratory of Population Genetics and Evolution of Vectors Mosquitoes at INPA in Manaus, Brazil.

A fragment, representing the DNA barcode region from *COI* gene, was amplified using the universal barcoding primers LCO 1490 and HCO 2198 [67], in a concentration of 10 μM. The amplification conditions were according to Motoki et al. [25]. A High Fidelity Platinum® Taq DNA Polymerase (Life Technologies) was included in all PCR reactions. The PCR products were electrophoresed in 1% agarose gel stained with GelRed™ Nucleic Acid Gel Stain (Biotium Inc., Hayward, USA) and observed under UV light to verify the sizes of the expected products and their quality. The amplified products were purified using PEG precipitation (20% polyethylene glycol 8000/2.5 M NaCl). Sequencing reactions were conducted for both DNA strands using the Big Dye Terminator Kit® and electro-injected into the automated sequencer ABI 3130xl Genetic Analyzer (Applied Biosystems, Thermo Fisher Scientific, Waltham, MA, USA) available at INPA.

The primers 5.8SF and 28SR were used to amplify the ITS2 region [7]. The amplification conditions are described in Motoki et al. [25]. A High Fidelity Platinum® Taq DNA Polymerase (Life Technologies) was used in all PCR reactions. The PCR products were electrophoresed in 1% agarose gel stained with GelRed™ Nucleic Acid Gel Stain (Biotium Inc., Hayward, USA), observed under UV light and purified by PEG precipitation. Sequencing reactions were carried out for both the DNA strands and electro-injected into an automated sequencer as described above. Due to logistic conditions, cloning of the specimens was not performed; therefore, the intragenomic variation could not be inferred.

### Statistical analysis of the DNA barcode and ITS2 markers

The sequences of the *COI* and ITS2 markers were automatically aligned in ClustalW [68] and edited in BioEdit v.7.2.5 [69] with the aid of Chromas Lite [70]. The consensus sequences of the *COI* gene generated a fragment size of 663 base pairs (bp), which was translated into amino acids to check for stop codons, pseudogenes or Numts. These sequences were compared with those available in GenBank (**S1 Table**) using the Basic Local Alignment Search Tool (BLAST) platform [71] available at the National Center for Biotechnology Information (NCBI) website (http://blast.ncbi.nlm.nih.gov/Blast.cgi). The consensus sequences of ITS2 generated a fragment size of 441 bp and were compared with the sequences of the *An*. *oswaldoi* and *An*. *konderi* species complexes available in GenBank. The access numbers of the sequences downloaded from GenBank are available in the supplementary file (**S1 Table).** The haplotypes of *COI* and ITS2 of this study are deposited in GenBank under access numbers: MG241899 –MG241941 and MG263750 –MG263759, respectively.

Both datasets (*COI* and ITS2) were checked for saturation levels using the DAMBE [72]. This analysis allows the identification of whether there was any saturation between transition and transversion rates in relation to genetic distances, which is informative for the phylogenetic inferences. From the *COI* dataset, the haplotype number was estimated using DnaSP v.5 [73] and TCS v.1.21 [74]. The genealogies among the haplotypes were generated using TCS software. Based on the parsimony criterion, a connection limit of 95% was established to investigate whether the species of the *An*. *oswaldoi* and *An*. *konderi* complexes formed a single “meta-population” represented by a single network or whether each species was represented by independent networks.

The phylogenetic relationships were inferred using the neighbor-joining (NJ) in MEGA v.6 [75], maximum parsimony (MP) in PAUP* v.4 [76], and maximum likelihood (ML) in Garli v.0.95 [77] algorithms, and Bayesian inference (BI) in MrBayes v.3.2.5 [78]. The nucleotide substitution model Kimura 2 Parameters (K2P) [79] were used in the NJ analysis with 2,000 replicates, whereas the ML and BI analyses were performed using the nucleotide substitution model HKY+I+G [80] previously selected with the Akaike Information Criterion (AIC) in the jModelTest [81]. This model assumes variation rates over the sites following a gamma distribution and a proportion of sites regarded as invariable. In the phylogenetic relationship using the ML, the branch supports (bootstrap) were assessed with 2,000 replicates. In the BI analyses, two simultaneous independent runs of the Markov Chain Monte Carlo (MCMC) were performed for 100 million generations, while sampling every 1,000 generations with a burn-in of 25%. Posterior probabilities (BPP) were used to assess nodal support.

The divergence time of the lineages (groups) was estimated using a relaxed lognormal clock with a Yule tree prior that assumes a constant lineage birth rate for each tree branch with a mutation rate of 2.3% for every million years for the *COI* gene [82]. This analysis was conducted in *BEAST v. 1.7 [83].

For the ITS2 dataset, the phylogenetic relationships were inferred using BI, and GTR + G was used as the nucleotide substitution model, which was previously selected with the Akaike Information Criterion (AIC) in the jModelTest [81]. The settings adopted herein were two simultaneous independent runs of the Markov Chain Monte Carlo (MCMC) for 100 million generations, sampling every 1,000 generations with a burn-in of 25%. BPP was used to assess nodal support.

A BI tree was also constructed with concatenated data (*COI*+ITS2). In this analysis, the datasets consisted of 27 sequences from the same individuals for each marker. The nucleotide substitution models used were GTR + I + G and GTR + G for *COI* and ITS2, respectively. In all phylogenetic analyses, *Anopheles* (*Nyssorhynchus*) *goeldii* and *Anopheles* (*Nyssorhynchus*) *marajoara* were used as outgroups. The former was chosen because it belong the same section (Albimanus) of *An*. *oswaldoi* [21], which they are more closely related species. The latter, because it belongs to a different section (Argyritarsis) from *An*. *oswaldoi* [21]; therefore, they can be more distant phylogenetically species. The generated trees were visualized and edited in FigTree v.1.3.1[84].

The intra and inter-specific genetic distances were calculated in MEGA v.6 [75] based on the nucleotide substitution model K2P. The presence or absence of the "barcoding gap" was evaluated by plotting the intra and inter-specific divergence values (K2P) in a frequency histogram.

#### Species delimitation

Molecular operational taxonomic units (MOTU’s) were identified according to the criterion of reciprocal monophyly based on the different phylogenetic approaches for each marker (*COI* and ITS2) and concatenated datasets (*COI* + ITS2). The species delimitation plugin was estimated in GENEIOUS R8 [85] to calculate Rosenberg’s *P*_AB_ value of the clades that had the highest support in BI. This test allows taxonomic distinction based on the null hypothesis of whether the monophyly has occurred by chance alone or if it is an artifact of undersampling [86]. To infer the limits between the species within the cryptic species complex, the Bayesian approach of General Mist of Yule Coalescent (bGMYC) [87] was used. This approach identifies uncertainties in the limits of the species as per the changes in the ramification rates in the phylogenetic tree when distinct populations contain several species [87]. These analyses were performed using the bGMYC SPLITS package [88] implemented in R v.3.0.1 (R Foundation for Statistical Computing, Vienna, Austria). The ultrametric trees were generated with BEAUTi and *BEAST v.1.7 [89] and used in these analyses.

Best Match (BM; sequences with smallest distance to query all conspecifics), Best Close Match (BCM; sequences with the smallest distance to query all conspecifics and within the 95^th^ percentile of all intraspecific distances) and All Species Barcodes (ASB; as in best match, but with all conspecific sequences topping the list of best matches) were used as evaluation criteria of species delimitation based on the *COI* barcode dataset used in this study. These algorithms were used to test the identification success and were carried out with TaxonDNA software [90]. Successful identification was determined based on the 1% standard threshold cut-off, as suggested by The Barcode of Life Data System as barcoding satisfactory gap to delimit cryptic species [91]. Automatic Barcode Gap Discovery (ABGD) analysis allowed partitioning of the DNA sequence datasets into clusters of similar taxa, establishing a range of maximum values of intraspecific divergence (*P*), without an *a priori* species hypothesis [92].

### Geographic distribution

Each identified species was plotted on the map. This analysis was implemented in Qgis v.2.4 [93] based on the data from this study as well as those available in the literature.

## Results

From the total anophelines collected in this study, 95 specimens were morphologically identified as *An*. *oswaldoi* s.l./*An*. *konderi* s.l. These specimens were collected at 18 sites in the Brazilian Amazon region. Of these, genomic DNA was isolated from 83 specimens (78 adults and 5 larvae), all (100%) of which were amplified for the DNA barcode region and 27 (32.50%) of which were amplified for the ITS2 region (**Table 1**). All phylogenetic analyses and the species delimitation bGMYC plugin, haplotype network and genetic distance confirmed the presence of five species in the Brazilian Amazon: two formally described species (*An*. *oswaldoi* s.s. and *An*. *konderi*) and three taxonomically non-described species (*An*. *oswaldoi* A, *An*. *oswaldoi* B and *An*. sp. nr *konderi*). The most abundant species were *An*. *konderi* (45.80%) and *An*. *oswaldoi* A (30.11%), followed by *An*. *oswaldoi* B (9.64%), *An*. sp. nr. *konderi* (8.43%) and *An*. *oswaldoi* s.s. (6.02%). *An*. *konderi* was recorded in Amapá, Amazonas, Pará and Rondônia; *An*. *oswaldoi* A in Amapá (recorded for the first time in this study), Amazonas and Pará; *An*. sp. nr. *konderi* in Acre, Amazonas and Rondônia; *An*. *oswaldoi* s.s. in Acre and Amazonas; and *An*. *oswaldoi* B only in Amapá (confirmed in this study). In addition, six sympatric occurrences are described below in the geographical distribution section.

### DNA barcode region

Eighty-three sequences generated a fragment length of 663 bp, with a large region of overlap (~ 626 bp), excluding the primer regions. This dataset did not reveal insertions or deletions, and the translated amino acid sequence did not have a stop codon, pseudogenes or Numts. Transitions were more common (90.80%) than transversions (9.20%). Analysis of the transition and transversion rates against the genetic distances (K2P) did not reveal saturation, suggesting that this dataset is informative for the phylogenetic analyses. The mean composition of the nucleotides was A = 29%, C = 15.5%, G = 16.5% and T = 39%, with contents of A+T = 68%.

**Fig 1:**
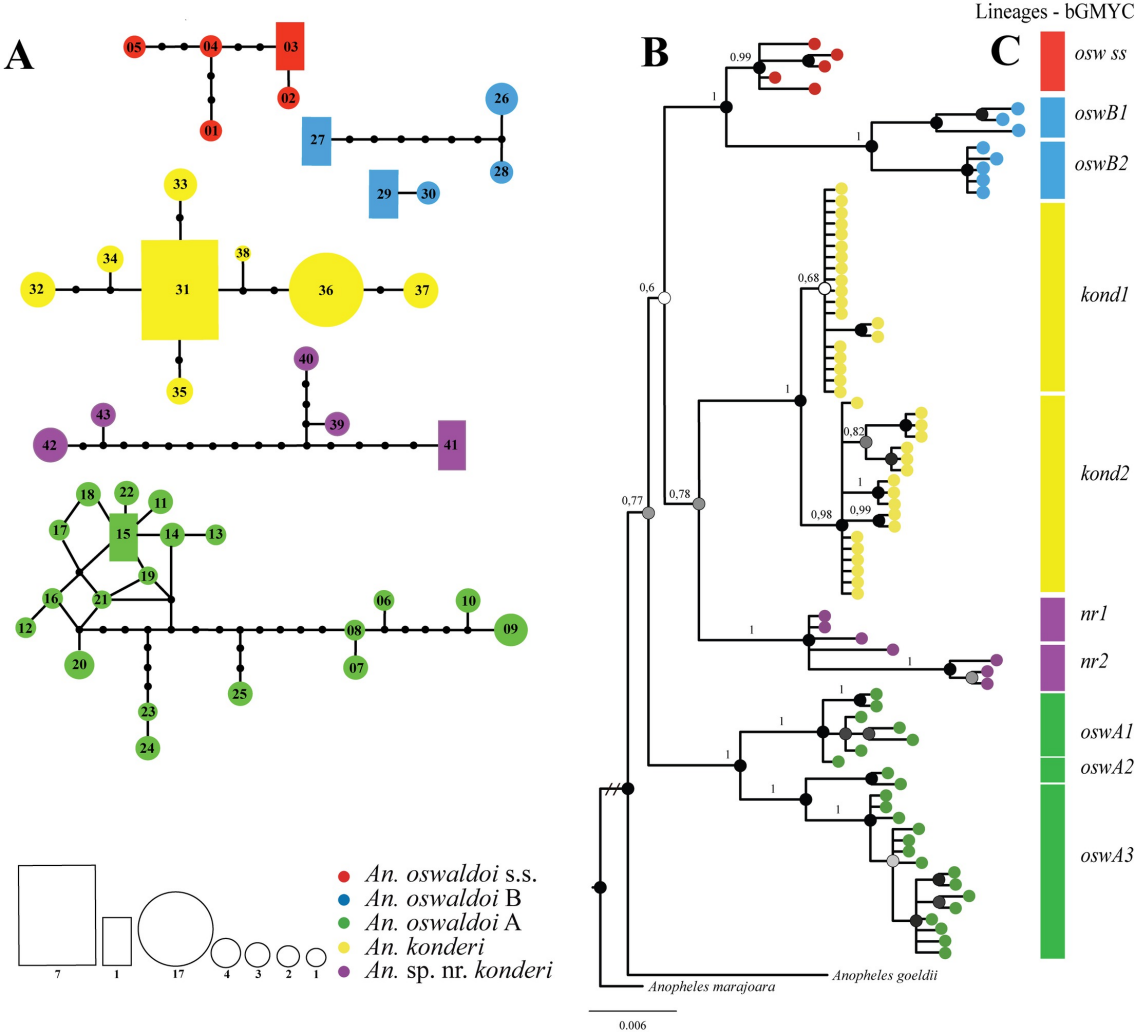
Genealogical and phylogenetic analyses obtained with the *COI* dataset for the five species of the Oswaldoi-Konderi complex. **Fig 1A.** Haplotype network generated with 95% confidence. Each species is represented by different colors. The circle sizes indicate the frequency of individuals observed in each haplotype. **Fig 1B.** Bayesian inference (BI) tree inferred with the HKY + I + G model. The supports of the branches, BPP (posterior probability), are indicated by circles in each node. Black circles > 0.95 BPP, gray circles ≤ 0.95 BPP and ≥ 0.70 BPP, and white circles < 0.70 BPP. *Anopheles goeldii* and *An*. *marajoara* were used as outgroups. **Fig 1C.** The bGMYC plugin indicates the separation of clades recovered in lineages. osw ss: *Anopheles oswaldoi* s.s.; oswB1: *An*. *oswaldoi* B (lineage 1); oswB2: *An*. *oswaldoi* B (lineage 2); kond1: *An*. *konderi* (lineage 1); kond2: *An*. *konderi* (lineage 2); nr1: *An*. sp. nr. *konderi* (lineage 1); nr2: *An*. sp. nr. *konderi* (lineage 2); oswA1: *An*. *oswaldoi* A (lineage 1); oswA2: *An*. *oswaldoi* A (lineage 2); oswA3: *An*. *oswaldoi* A (lineage 3).

Eighty-three sequences generated 43 haplotypes, of which 28 (65.12%) were singletons and 15 (34.88%) were shared (**S2** and **S3 Tables**). *An*. *oswaldoi* A, the second most frequent species, had the largest number of haplotypes (H6 to H25). **Fig 1A** shows the six disconnected networks, in which each haplotype network represents one species: *An*. *oswaldoi* s.s. (in red), *An*. *oswaldoi* B (in blue), *An*. *oswaldoi* A (in green), *An*. *konderi* (in yellow) and *An*. sp. nr. *konderi* (in purple). *An*. *oswaldoi* B, however, had two disconnected haplotype networks. This situation occurred because the two haplotypes (H26 and H29) observed sympatrically in Serra do Navio, as well as the two haplotypes (H28 and H30) from Ferreira Gomes, were not connected to each other, but haplotypes H29 and H30 in these localities connected with each other, resulting in a second network. Misidentification cannot be the cause of this result because these haplotypes were clustered into the clade represented by *An*. *oswaldoi* B (**Fig 1B**). Thus, this finding may indicate undersampling or sampling gaps, necessitating future investigations. The network represented by *An*. *oswaldoi* A revealed many missing haplotypes and reticulations, likely suggesting undersampling or sampling gaps or genetic subdivision, which also need additional investigation.

The BI tree (**Fig 1B**) and NJ tree (**S1 Fig**) generated identical topologies with two main clades. In the BI tree, the apical clade (BPP: 0.60) was subdivided into two subclades. Subclade 1 (BPP: 1.0) clustered *An*. *oswaldoi* s.s. and *An*. *oswaldoi* B. Subclade 2 (BPP: 0.78) clustered *An*. *konderi* and *An*. sp. nr *konderi*. The most basal clade (BPP: 1.0) was formed by *An*. *oswaldoi* A to generate the *An*. *oswaldoi* s.l. paraphyletic complex. Within this clade, three strongly supported subgroups (BPP: 1.0) were observed for *An*. *oswaldoi* A. Subgroup oswA1 clustered samples of Pitinga, Serra do Cachorro and Calçoene, oswA2 grouped samples of Mata Fome and Santa Bárbara, and oswA3 clustered samples of Lábrea and Coari (see **Table 1**). All clades and subclades were mutually monophyletic.

The lineage sorting in bGMYC identified five taxa, supporting the grouping generated in the BI tree, and confirmed two subgroups for *An*. *oswaldoi* B (oswB1 and oswB2) and three for *An*. *oswaldoi* A (oswA1, oswA2 and oswA3). The bGMYC also created two subgroups for each species of *An*. *konderi* and *An*. sp. nr. *konderi*, but had moderate and high support, respectively (BPP: 0.68; 1.0).

**Fig 2:**
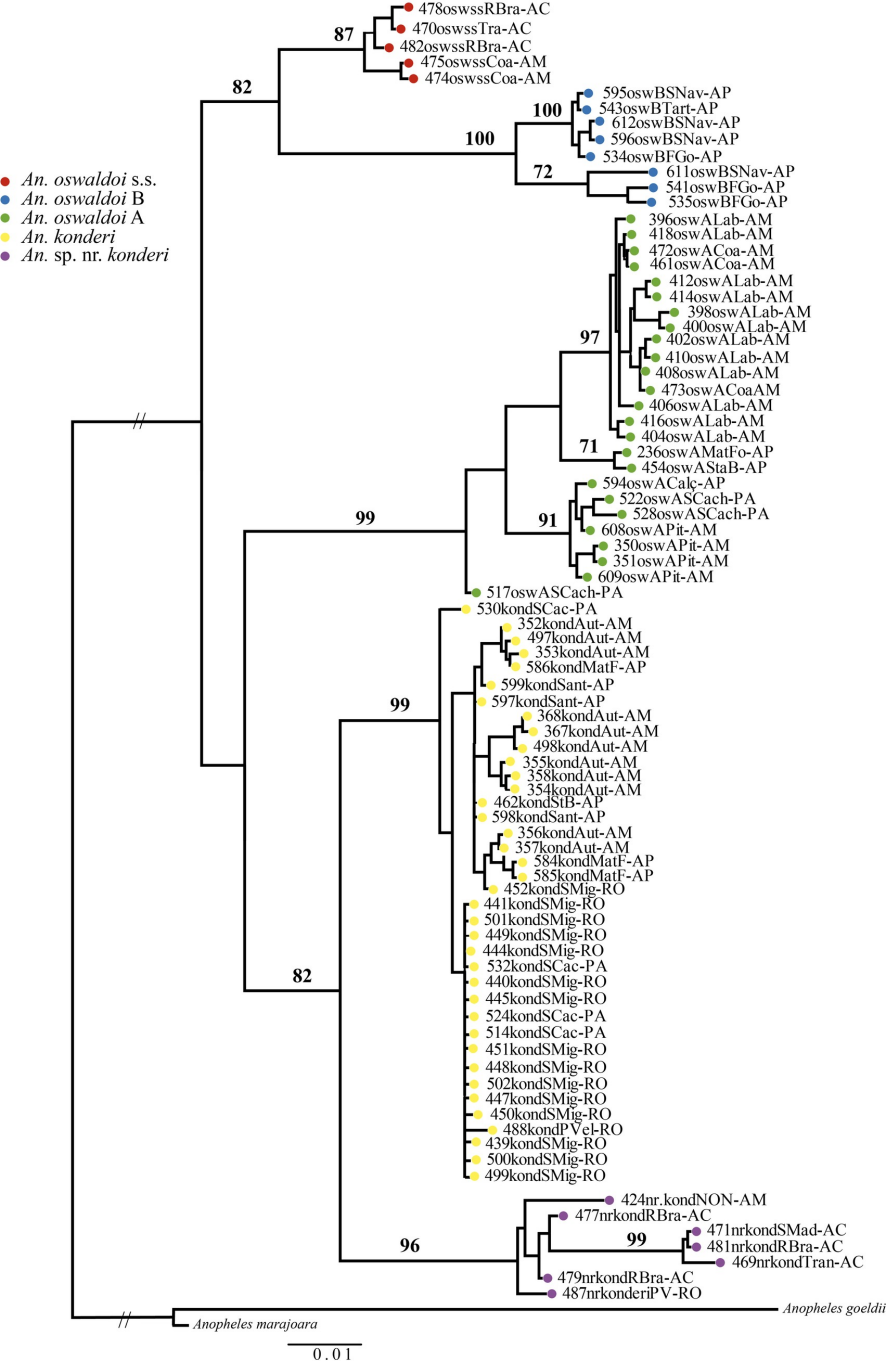
Maximum-likelihood (ML) tree generated with the *COI* dataset for five species of the Oswaldoi-Konderi complex. Tree inferred with the HKY + I + G model. The bootstrapping values (2,000 replicates) are indicated above of the branches.

The ML tree (**Fig 2**) generated a topology with better resolution than the maximum parsimony (MP) tree (**S2 Fig**), which recovered only the terminal clades but were monophyletic and strongly supported. Although the ML tree also revealed two major Clades, I and II, it also showed a distinct topology from the NJ and BI trees. Clade I (bootstrapping support: 0.82) consisted of two subclades, *An*. *oswaldoi* s.s. and *An*. *oswaldoi* B, indicating that they are phylogenetically closely related species. Clade II (bootstrapping support: 0.82) consisted of two subclades. The most basal subclade clustered samples of *An*. *oswaldoi* A, whereas the other subclade clustered *An*. *konderi* and *An*. sp. nr. *konderi*. This finding suggests that *An*. *oswaldoi* A is phylogenetically more closely related to *An*. *konderi* and *An*. sp. nr. *konderi* than to *An*. *oswaldoi* s.s. and *An*. *oswaldoi* B, again suggesting that the *An*. *oswaldoi* complex is paraphyletic. All clades and subclades were reciprocally monophyletic.

**Table 2** shows the means of the intra and interspecific genetic distances (K2P) estimated with the *COI* dataset among the five species. Mean intraspecific genetic distances ranged from 0.5% (*An*. *konderi*) to 1.4% (*An*. sp. nr. *konderi*), whereas the interspecific genetic distances ranged from 3.8% (between *An*. *konderi* and *An*. sp. nr. *konderi*) to 6.2% (between *An*. *oswaldoi* A and *An*. *oswaldoi* B). *An*. *oswaldoi* s.s. was closely related to *An*. *oswaldoi* B and *An*. *konderi* (both 3.9%). *An*. *oswaldoi* A had the largest range of values of intraspecific genetic distances (from 0% to 2.9%; mean 1.3%±0.1%), followed by *An*. *oswaldoi* B and *An*. sp. nr. *konderi*, with identical range values (from 0% to 2.3%; means of 1.2%± 0.8% and 1.4%± 0.8%, respectively). *An*. *konderi* and *An*. *oswaldoi* s.s. had the lowest mean values (0.5%±0.4% and 0.7%±0.3%, respectively).

**Table 2:** Mean intra and interspecific genetic distances (K2P) obtained with the *COI* dataset for the species of the Oswaldoi-Konderi complex.

Species	oswss	oswA	oswB	kond	nr.kond
*An*. *oswaldoi* s.s.	**0.007**				
*An*. *oswaldoi* A	0.052	**0.013**			
*An*. *oswaldoi* B	0.039	0.062	**0.012**		
*An*. *konderi*	0.039	0.052	0.053	**0.005**	
*An*. sp. nr. *konderi*	0.048	0.054	0.059	0.038	**0.014**

**oswss:** *An*. *oswaldoi* s.s., **oswA:** *An*. *oswaldoi* A, **oswB:** *An*. *oswaldoi* B, **kond:** *An*. *konderi*, **nr.kond:** *An*. sp. nr. *konderi*. K2P: Kimura 2 Parameters. Values in **bold** represent intra-specific distances.

**S4 Table** portrays the genetic distances among the lineages of *An*. *oswaldoi* A, *An*. *oswaldoi* B and *An*. sp. nr. *konderi*. The three lineages observed for *An*. *oswaldoi* A ranged from 1.5% (oswA2 and oswA3) to 2.4% (oswA1 and oswA2), whereas between the two of *An*. *oswaldoi* B was at 2.1% and between the two *An*. sp. nr. *konderi* at 1.9%. Nonetheless, the bGMYC plugin based on the genetic distances separated all the sequences in ten lineages, but the significant values were observed only between oswA1 and oswA2, oswA1 and oswA3, and between oswB1 and oswB2.

**S3 Fig** portrays a histogram of the mean intra and interspecific genetic distances among the five species based on the ABGD analysis. The barcode gap was well below (0.4%) the threshold value of 1% to delimit cryptic species [94]. This result can be attributed to the presence of distinct lineages within *An*. *oswaldoi* A and *An*. *oswaldoi* B.

### ITS2 region

Twenty-seven sequences had lengths that varied from 441 bp to 511 bp. **S5 Table** shows ten haplotypes and their respective variable and fixed sites, and the indels observed in the five species. The dataset shows 20 variable sites distributed between 194 and 497 sites. The indels were verified at positions 325, 351, 471, 479 and 497, allowing the distinction of each analyzed species. *An*. *oswaldoi* s.s. showed a transversion at position 393 (A-C) and a transition at position 432 (C-T). *An*. *oswaldoi* A showed a transition at position 194 (A-G), and *An*. *oswaldoi* B had a deletion at position 351, four transversions at positions 389 (C-A), 393 (A-G), 397 (A-C) and 453 (G-C), and a transition at position 462 (G-A). *An*. *konderi* only showed a transition at position 440 (C-T) and *An*. sp. nr. *konderi* had an insertion at position 325 (G). *An*. *oswaldoi* B and *An*. *konderi* also had insertions (adenosine) at this site (325).

**Fig 3:**
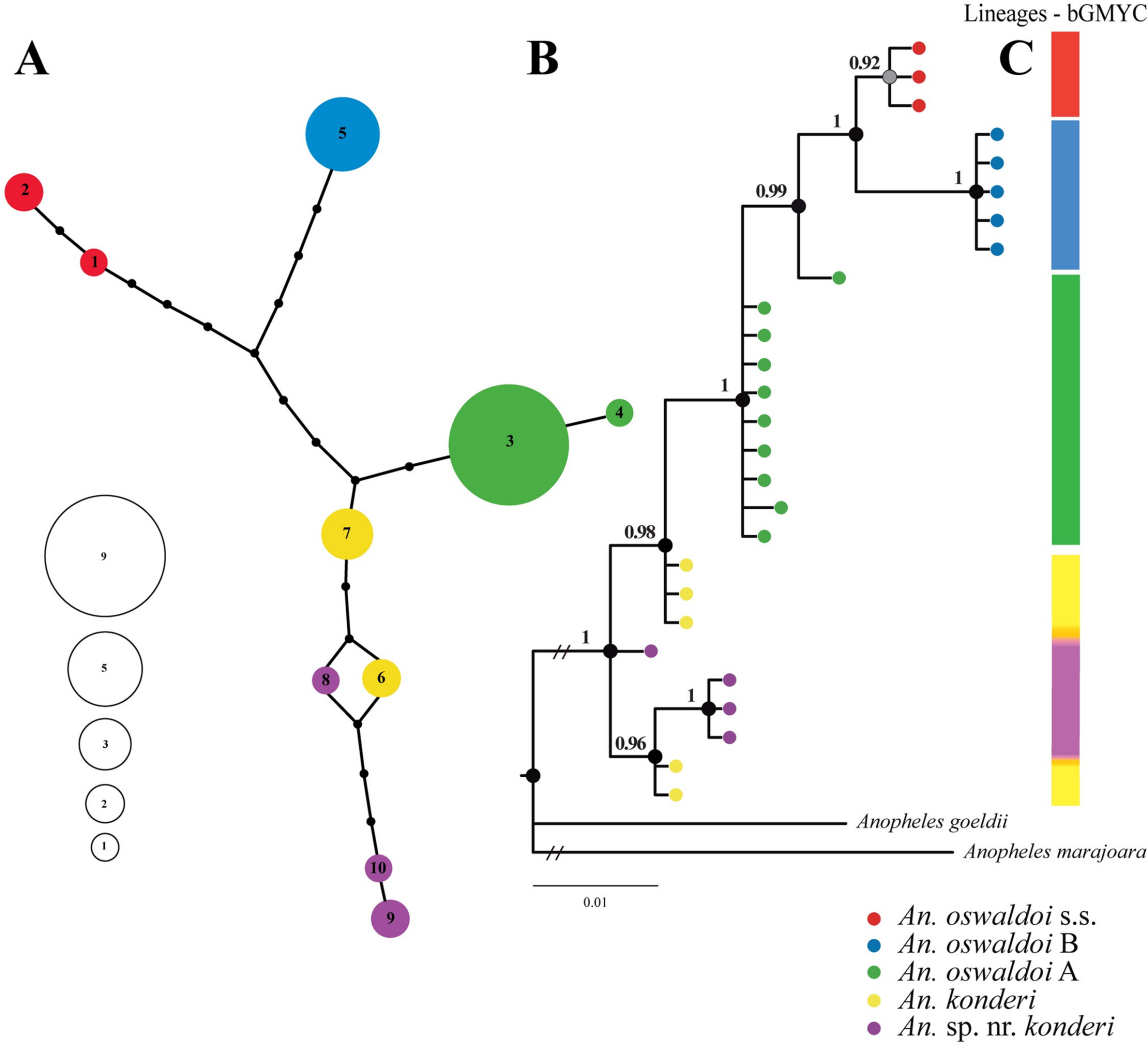
Genealogical and phylogenetic analyses obtained with the ITS2 dataset for the species of Oswaldoi-Konderi complex. **Fig 3A.** Haplotype network generated with 95% confidence. Each species is represented by a different color. The circle sizes indicate the frequency of individuals observed in each haplotype. **Fig 3B.** Bayesian inference (BI) tree inferred using the GTR + G model. The supports on the branches, BPP (posterior probability), are indicated by circles in each node. Black circles > 0.95 BPP, gray circles ≤ 0.95 BPP and ≥ 0.70 BPP, and white circles < 0.70 BPP. *Anopheles goeldii* and *An*. *marajoara* were used as outgroups. **Fig 3C.** The bGMYC plugin indicates the lineages.

The ITS2 dataset indicated saturation between transition (*Ts*) and transversion (*Tv*) rates in the range of genetic distances from 3.0% to 3.5%. The genetic distance values obtained with this marker were below this range; hence, it is phylogenetically informative. The mean nucleotide composition was, A = 28%, C = 27%, G = 25% and T = 20%, with the content of G+C = 52%. From the ten haplotypes (**S6 Table**), three were observed in *An*. sp. nr. *konderi* (H8, H9, H10), followed by *An*. *oswaldoi* s.s., *An*. *oswaldoi* A and *An*. *konderi* with two haplotypes each (n = 2 each), and *An*. *oswaldoi* B with one haplotype (H5). Unlike the *COI* dataset, all haplotypes were connected in a unique network (**Fig 3A**), and at least three mutational events separated the species except in *An*. *konderi* and *An*. sp. nr. *konderi*, where haplotypes 6 and 8 formed a reticulation likely indicating homoplasy. The BI tree (**Fig 3B**) was well resolved for *An*. *oswaldoi* s.s. and *An*. *oswaldoi* B, in which all sequences of each species were clustered together. Nonetheless, the sequences of *An*. *oswaldoi* A, *An*. *konderi* and *An*. sp. nr. *konderi* did not clearly separate, forming a polytomy, but the groups were highly supported. The sequences of *An*. *konderi* were clustered in distinct groups, both highly supported, indicating two lineages within *An*. *konderi*. The low number of sequences analyzed for this marker and the probable existence of intragenomic variation in these species likely contributed to this result.

In addition, analyses of Rosenberg’s *P*_AB_ using the *COI* dataset indicated significant values for the five species (*p* < 0.05), suggesting that they represent biological species. Nevertheless, for the *COI*, the BM, BCM and ASB values correctly identified all examined sequences (83: 100%) using the best matching threshold of 2%, confirming the existence of five biological species.

The bGMYC was also tested in the two datasets to delimit evolutionary discrete lineages, which are represented by the color bar in the *COI* (**Fig 1C**) and ITS2 (**Fig 3C**) trees, adopting a probability threshold (*p* = 0.95). These results indicated that there were from 5 to 10 lineages for the *COI* and from 2 to 5 lineages for the ITS2. This analysis matched with Rosenberg’s *P*_AB_, supporting the identification of five species within the complex.

**Table 3** shows the mean intra and interspecific genetic distances (K2P) obtained in the ITS2 dataset, in which the values were lower and none corresponded to those of the *COI* dataset. Mean intraspecific genetic distances ranged from 0% (*An*. *oswaldoi* A and *An*. *oswaldoi* B) to 0.7% (*An*. *konderi*). The mean interspecific genetic distances ranged from 0.7% (between *An*. *oswaldoi* A and *An*. *konderi*) to 2.1% (between *An*. *oswaldoi* s.s. and *An*. *oswaldoi* B).

**Table 3:** Mean intra and interspecific genetic distances (K2P) obtained with the ITS2 dataset for the species of the Oswaldoi-Konderi complex.

Species	oswss	oswA	oswB	kond	nr.kond
*An*. *oswaldoi* s.s.	**0.003**				
*An*. *oswaldoi* A	0.012	**0.000**			
*An*. *oswaldoi* B	0.021	0.017	**0.000**		
*An*. *konderi*	0.015	0.007	0.017	**0.007**	
*An*. sp. nr. *konderi*	0.019	0.012	0.020	0.009	**0.002**

**oswss:** *An*. *oswaldoi* s.s., **oswA:** *An*. *oswaldoi* A, **oswB:** *An*. *oswaldoi* B, **kond:** *An*. *konderi*, **nr.kond:** *An*. sp. nr. *konderi*. K2P: Kimura 2 Parameters. Values in **bold** represent intra-specific distances.

### Delimitation of species and estimates of divergence time

**Fig 4:**
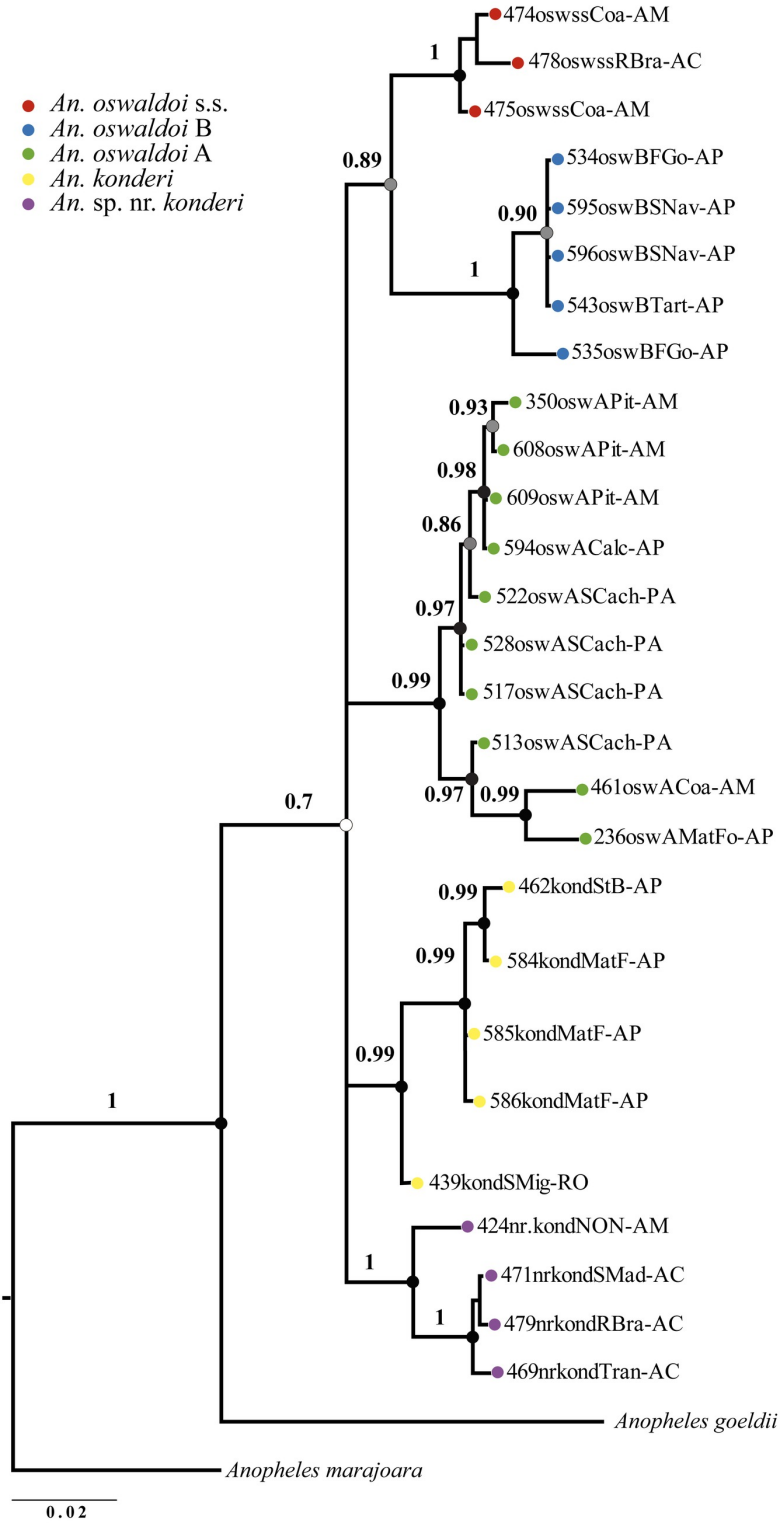
Bayesian inference tree generated with the concatenated data (*COI*+ITS2) for the species of the Oswaldoi-konderi complex. Tree inferred using the GTR + I + G and GTR + G models for *COI* and ITS2, respectively. The supports of the branches, BPP (posterior probability), are indicated by circles in each node. Black circles > 0.95 BPP, gray circles ≤ 0.95 BPP and ≥ 0.70 BPP, and white circles < 0.70 BPP. *Anopheles goeldii* and *An*. *marajoara* were used as outgroups.

The BI tree with concatenated data (*COI* + ITS2) generated a topology with five clades representing five species (**Fig 4**). However, only the clades represented by *An*. *oswaldoi* s.s. and *An*. *oswaldoi* B (BPP: 0.89) were well resolved. The remaining clades formed polytomies and had moderate support (BPP: 0.70). Although, it was not possible to establish relationships among the five clades, all terminal branches were strongly supported and monophyletic (*An*. *oswaldoi* s.s., *An*. *oswaldoi* B, *An*. sp. nr. *konderi* with BPP: 1.0, *An*. *oswaldoi* A and *An*. *konderi* with BPP: 0.99).

**S4 Fig** shows the BI tree generated with all sequences of the barcode region (this study+GenBank) for the five species. After adjustment and trimming, the sequences had a length of 641 bp. In this analysis, the three lineages of *An*. *oswaldoi* A were maintained. Interestingly, lineage oswA1 (strongly supported, BPP: 0.99), represented by sequences from Pitinga, Serra do Cachorro and Calçoene (north of the Amazonas river), did not cluster with any sequences of *An*. *oswaldoi* A from GenBank. The lineage oswA2 (moderately supported, BPP: 0.84), represented by sequences from Mata Fome and Santa Barbara (north of the Amazonas river), were grouped into a subclade formed by the sequences from Bom Jesus de Tocantins, state of Pará, and Peixoto de Azevedo, state of Mato Grosso (south of the Amazonas river) downloaded from GenBank. Lineage oswA3 (strongly supported, BPP: 0.95), represented by sequences from Lábrea and Coari (south Amazonas river), were clustered into a subclade formed by the sequences from Acrelândia, state of Acre, Peixoto de Azevedo, state of Mato Grosso (south of Amazonas river), and Leticia, in Colômbia, downloaded from GenBank. Thus, lineage oswA1 was observed for the first time in the present study and may represent a new subclade within *An*. *oswaldoi* A.

**Fig 5:**
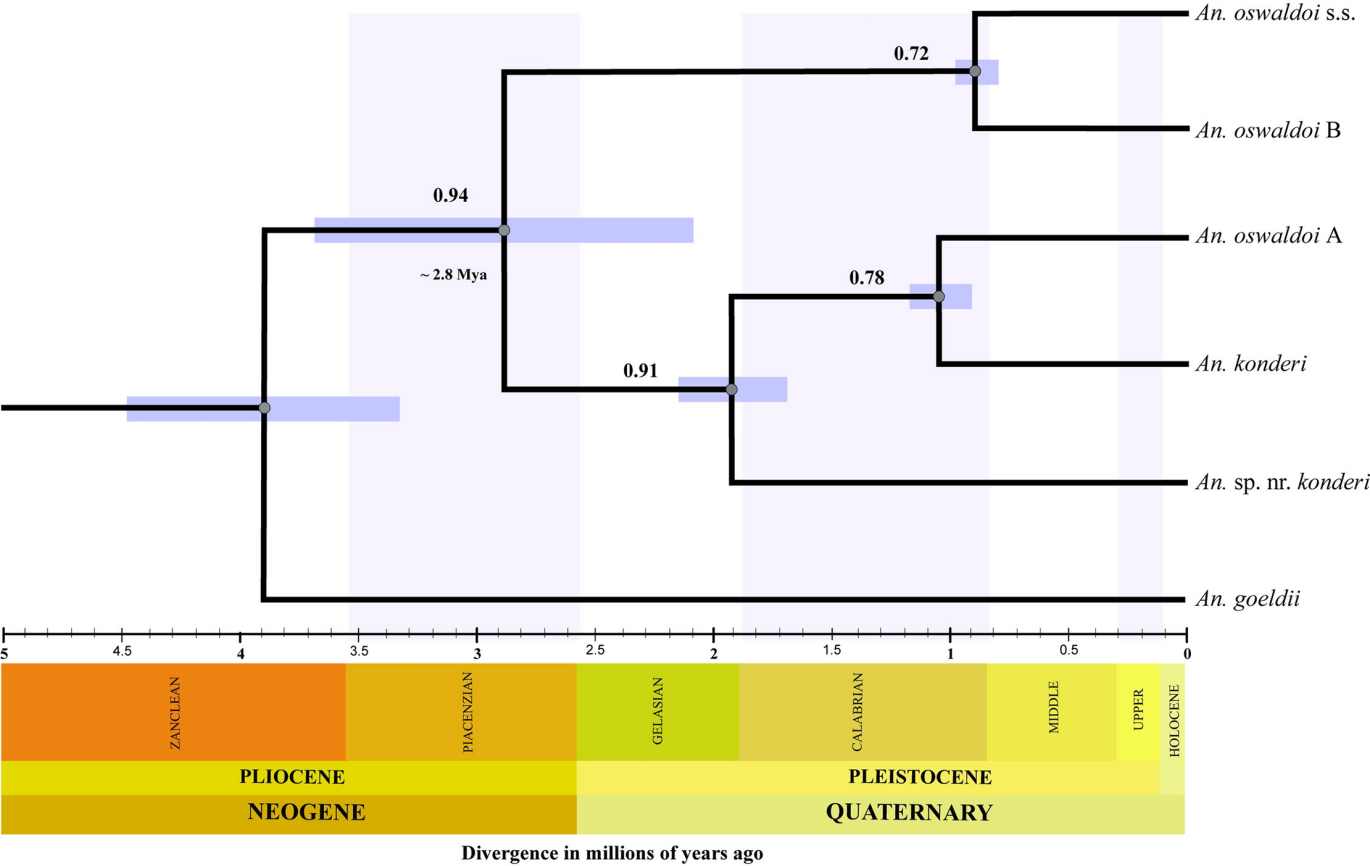
Species tree topology generated with the maximum credibility of a multispecies coalescence model. Analysis performed with concatenated data (*COI* + ITS2) using molecular dating based on the *COI* gene and assuming a relaxed clock with a mutation rate of 2.3% every Mya. Estimates of the mean divergence time of the nodes are reported with intervals of 95% HPD (highest posterior density) and are represented as bars in the nodes. The supports on the branches are indicated by gray circle from ≤ 0.95 BPP to ≥ 0.70 BPP.

The species tree with concatenated data (**Fig 5**) estimated the phylogenetic relationship and divergence time of the five species. This tree showed two major clades: Clade I, clustered *An*. *oswaldoi* s.s. and *An*. *oswaldoi* B (BPP: 0.72), and Clade II, clustered *An*. *konderi* and *An*. *oswaldoi* A in one branch, which were more closely related (BPP: 0.78), and *An*. sp. nr. *konderi* (BPP: 0.91) more basally in another branch. The divergence time indicated that the most recent cladogenesis event occurred between *An*. *oswaldoi* s.s. and *An*. *oswaldoi* B and was dated ~ 0.9 (0.7 to 1.0) Mya, followed by cladogenesis between *An*. *oswaldoi* A and *An*. *konderi* ~ 1.2 (0.8 to 1.3) Mya. The clade formed by *An*. *oswaldoi* A/*An*. *konderi* and *An*. sp. nr. *konderi* was dated ~ 1.8 (1.3 a 2.2) Mya. Finally, the node that indicates the oldest diversification event between the two major clades was dated ~ 2.8 (1.4 to 5.0) Mya. These time periods fall between the Neogene (Pliocene) and the Quaternary (Pleistocene) epochs. These estimates are a rough approximation of the evolutionary history of the complex.

### Geographic distribution

**Fig 6:**
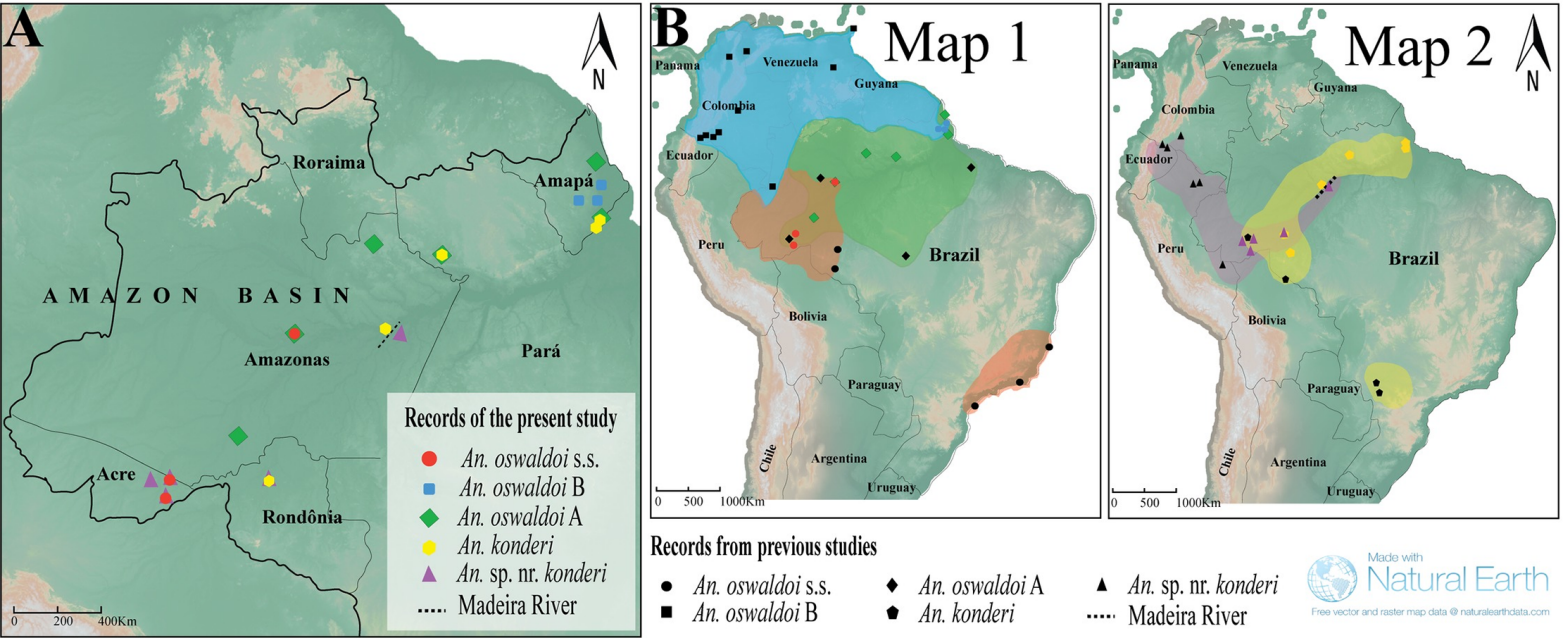
Map of the geographic distribution of the five species of the Oswaldoi-Konderi complex. **Fig 6A.** Distribution of species collected in the present study. Each species is illustrated with different symbols and colors. **Fig 6B.** General distribution including the data from this study (colored symbols) and the records available in the literature (black symbols). Polygons roughly indicate the records of each species and the sampling gaps. Map 1 represents the geographic distribution of the species of the *Anopheles oswaldoi* complex; Map 2 represents the geographic distribution of the species of the *Anopheles konderi* complex. Map adapted from the USGS National Map Viewer (public domain): http://viewer.nationalmap.gov/viewer/ for illustrative purposes.

**Fig 6** shows the maps with the geographical distribution of the five species based on the records of the present and previous studies. In this study, *An*. *oswaldoi* s.s. was recorded in three of the 18 sampled localities, and it was restricted to the occidental Brazilian Amazon (Rio Branco, Acrelândia and Coari). It was sympatric with *An*. *oswaldoi* A and *An*. sp. nr. *konderi* in the states of Amazonas and Acre, respectively. *An*. *oswaldoi* B was captured from three sites of the state of Amapá (Ferreira Gomes, Tartarugalzinho and Serra do Navio) and was not sympatric with any other species. *Anopheles oswaldoi* A was the second most collected species and was recorded in six localities. It was sympatric with *An*. *konderi* in Macapá and Serra do Cachorro, and with *An*. *oswaldoi* s.s. in Coari. *Anopheles konderi* had the largest distribution in the Brazilian Amazon and was recorded in seven localities, but it was absent in Coari, its type locality. It was sympatric with *An*. *oswaldoi* A, as described above, and with *An*. sp. nr. *konderi* in Porto Velho. *An*. sp. nr. *konderi* was recorded in five locations in the Brazilian Amazon. It was recorded in Nova Olinda do Norte, whereas *An*. *konderi* was recorded in Autazes; the aforementioned sites are situated on opposite banks of the river Madeira in the state of Amazonas.

## Discussion

### Molecular taxonomy of *An*. *oswaldoi* s.l. and *An*. *konderi* s.l.

Based on the two markers used in this study, the *COI* barcode was sufficient for identifying the five species: *An*. *oswaldoi* s.s., *An*. *oswaldoi* B, *An*. *oswaldoi* A, *An*. *konderi* and *An*. sp. nr. *konderi*, confirming the findings of previous studies [7,13–16]. The ITS2 fragment alone was not sufficient to separate the following species: *An*. *konderi*, *An*. sp. nr. *konderi* and *An*. *oswaldoi* A, which were not monophyletic in the BI analyses. Although this nuclear marker alone did not resolve the phylogenetic relationships among these species, its use in the concatenated analyses delimited the five strongly supported groups. Previous studies have mentioned that the use of unique molecular marker is not sufficient for species separation [17,95], especially when they have recently diverged. A similar situation was reported in the Strodei subgroup, where the *COI* gene (barcode region) alone was unable to separate the species *An*. *arthuri* and *An*. *albertoi*, which are morphologically distinct, but they were separated successfully with the use of concatenated markers (*COI*+ITS2) [17].

Despite the criticism regarding the use of unique marker [17,95], such as a DNA barcode for species identification [96], the findings of this study revealed that the DNA barcode (*COI* gene) was efficient for separating all the species of the Oswaldoi-Konderi complex, as well as the nine species and three lineages of the Albitarsis complex [97,98] and the species of the Nuneztovari complex [10]. Thus, the DNA barcode can also be used as a signal to identify atypical specimens for further comprehensive taxonomic investigations [99]. Nonetheless, although this marker has successfully separated the five groups of the Oswaldoi-Konderi complex, the concatenated analyses that included the nuclear marker, did not allow us to more accurately determine the phylogenetic relationships of the five species of this study. This finding can be attributed to the low number of sequences in the ITS2 dataset.

In the ITS2 region, differences in length and fixed substitutions among sequences are considered proof of lineage splitting, especially if the lineages are geographically co-distributed [100]. The effect of concerted evolution in homogenizing the multiple copies at the intra-population level [101] leads to a tendency toward fast divergence of the sequences at the inter-population level, making ITS2 an appropriate marker for the separation of recently diverged species [102], as has been efficiently demonstrated in anopheline species [13,15,25,103–107]. The present findings were no different; this marker showed differences in length and fixed substitutions in the five species, allowing them to be separated (**S5 Table**), regardless of whether they were collected in sympatry or in allopatry, and it should reflect the status of distinct species within the Oswaldoi-Konderi complex. Among the five species, *An*. *oswaldoi* B had the largest number of mutations that allowed differentiating it from the other species, but only one haplotype was observed. The absence of variation in *An*. *oswaldoi* B may be because it was collected in a small geographic area and/or due to the small sample size. However, unlike the observation of Ruiz-Lopez et al. [16], when this marker was analyzed separately in this study, it did not generate well resolved phylogenies, but it indicated the three species of *An*. *oswaldoi* complex more closely related and two lineages within *An*. *konderi*. Further studies with larger number sequences for ITS2 and intra-individual cloning analysis may clarify these relationships.

In the present study, the ranges of *COI* intra and inter-specific genetic distances among the five taxa were very similar to those reported by Ruiz-Lopez et al. [16]. The pairwise distance values among the species of the Oswaldoi-Konderi complex were greater than 3%, and the phylogenetic trees with this marker were well resolved, forming strongly supported reciprocally monophyletic clades, unlike the results obtained for the Nuneztovari complex with minor differentiation (distances = 2.0% to 2.7%) [10]. Comparison of the results and species tree of this study with the Nuneztovari complex data [10] suggests an earlier initiation of the diversification process in the Oswaldoi-Konderi complex. Additionally, although the barcoding gap was lower than recommended (0.4%) [108], the 2% cut-off value used in the TaxonDNA analysis was satisfactory to safely delimit the species [91]. This result may be attributed to the presence of distinct genetic lineages within *An*. *oswaldoi* A and within *An*. *oswaldoi* B, for which the genetic distances were greater than 2% (2.1–2.4%) between lineages. Hebert et al. [109] argued that the barcoding gap allows the discovery of new species. Among the recently diverged species, however, there might be overlapping of intra and inter-specific distances, mainly due to the separation of incomplete lineages (ancestral polymorphism, introgression) [91,110]. Furthermore, other factors may affect the barcoding gap such that it is low or absent, e.g., undersampling or sampling gaps, populations with large effective size, significantly structured populations or highly divergent haplotypes within the populations due to the loss of the intermediary [91,111]. The overlap between intra and inter-specific distances in the *An*. *strodei* subgroup [17] was interpreted as an incomplete separation of lineages.

### Phylogenetic relationships and divergence estimation

The phylogenetic reconstruction (NJ, ML and BI) generated with the DNA barcode recovered five reciprocally monophyletic clades (from moderately to strongly supported) that corresponded to the five species, in accordance with the bGMYC, TCS network and genetic distance analyses. The phylogenetic relationships suggested that two pairs very closely related species correspond to *An*. *oswaldoi* s.s. and *An*. *oswaldoi* B and to *An*. *konderi* and *An*. sp. nr. *konderi*. Nonetheless, in the NJ and BI trees, only the specimens of *An*. *oswaldoi* A were clustered in one clade (basal), whereas in the ML tree the specimens of *An*. *oswaldoi* A were clustered in the main clade together with *An*. *konderi* and *An*. sp. nr. *konderi*. However, the *An*. *oswaldoi* complex was observed to be paraphyletic in all analyses, which was similar to the result reported by Ruiz-Lopez et al. [16]. Thus, the Oswaldoi complex (with three species) and the Konderi complex (with two species) may be considered a single complex named the Oswaldoi-Konderi complex, as suggested by Ruiz-Lopez et al. [16]. This phylogenetic relationship conforms to the genetic distances obtained in this study and the high morphological similarity among these species [33,34,105].

In the present study, the clade representing *An*. *konderi* was subdivided in two subgroups; however, both were weakly supported (**Figs 2 and 4**) and had low and non-significant genetic distances using the delimitation of species bGMYC plugin. One subsubgroup clustered the specimens from Amapá and Amazonas, and the other clustered those from Rondônia and Pará. Motoki et al. [25] analyzed the samples of the *An*. *konderi* complex from Acre, Amapá, Rondônia and Paraná and suggested three species in this complex. In the present study, the BI tree (**S4 Fig)** generated with all sequences (this study + GenBank) indicated that the samples of Rondônia and Paraná, inferred as new species by Motoki et al. [25], clustered into the clade of *An*. *oswaldoi* s.s.(**S4 Fig**). Thus, the specimens of Rondônia and Paraná [25] may in fact be *An*. *oswaldoi* s.s., thereby refuting the hypothesis of a new species within the *An*. *konderi* complex. If this is correct, the geographic distribution of *An*. *oswaldoi* s.s. may also be expanded to the state of Paraná in southern Brazil, in addition to Rio de Janeiro, Espírito Santo, São Paulo, Acre, Amazonas and Rondônia.

As observed in this study, the *COI* dataset was analyzed using the bGMYC, and this analysis separated it into ten lineages corresponding to five species. Within the species, this analysis was significant between the lineages of *An*. *oswaldoi* A (oswA1 and oswA2; oswA1 and oswA3) and the lineages of *An*. *oswaldoi* B (oswB1 and oswB2) (**S4 Table**). The lineages oswB1 and oswB2, however, may be an artifact of undersampling. A genetic distance greater than 2% between lineages of *An*. *oswaldoi* A could be indicative of incipient species [61]. If these lineages really represent incipient species, this level of differentiation could be the result of the Amazonas River acting as barrier to gene flow. Nevertheless, although the genetic distances were greater than 2% between oswA1 and oswA2 and between oswA1 and oswA3, and were significant according to the bGMYC analysis, the TCS network connected all haplotypes, indicating that these lineages may comprise a single species. This situation can be clarified with a comprehensive study of population genetics that includes more samples from each of the analyzed localities, as well as additional sampling to reduce gaps, which would elucidate the nature of the genetic variation of *An*. *oswaldoi* A or address questions about its taxonomic status, which has not yet been formally described.

In contrast, the nuclear dataset (ITS2) despite showing differences in length and fixed substitutions among the sequences that permitted separation of the species of this complex, the divergence, TCS network and phylogenetic analyses did not provide a clear resolution. Very low divergence (0.7–2.1%) was observed between the species depending on the species pairs compared. In the TCS network, all the haplotypes were connected in a unique network, and the phylogenetic analyses were not well resolved. Hence, the relationships between the species could not be inferred. These findings may be the result of intragenomic variation and the analysis of only a few sequences. McKeon et al. [97] and Moreno et al. [12] reported very similar findings for other anophelines with this marker.

Nuclear DNA markers are expected to provide older demographic information than mtDNA markers because dissimilar effective population sizes can affect coalescence time estimations. The mitochondrial loci in most species have a shorter expected coalescence time than nuclear loci (only one-fourth of the effective population size), and thus there is a greater probability that the mitochondrial gene tree will accurately reflect the species tree. As discussed above, the ITS2 marker showed different lengths between putative species. The characteristic mutation replacement of this marker (elevated number of indels and low frequency of replacement mutations) may be more useful for species-specific PCR diagnostics in species complex members than for phylogenetic inference [112].

The species tree topology (**Fig 5**) suggested that the diversification process of the Oswaldoi-Konderi complex likely occurred between the Neogene (Pliocene) and Quaternary (Pleistocene). During the Pliocene, large lakes formed the Amazon landscape due to the marine incursions likely caused by the orogeny of the Andes Mountain and the rise in sea level [113]. This event would have isolated three regions with higher relief, as represented by the Guiana and Brazilian Shields, and the Andes, which have been considered an explanation for the diversification processes in several animal groups, including insects [113]. In insects, this event has been hypothesized to explain the distribution patterns and divergence time in the *An*. *albitarsis* and *An*. *nuneztovari* complexes and *An*. *darlingi* [3,4], in the *Lutzomyia longipalpis* complex [114], and between *Rhodinus prolixus* and *Rhodinus robustus* [115], among others.

However, several discussions and hypotheses have been proposed to explain the phylogeographic patterns that occurred in South America during the Miocene. Turchetto‐Zolet et al. [116] argued that the climatic oscillations during the Pleistocene as well as the orogeny events that occurred during Miocene/Pliocene helped to shape the diversity and distribution patterns of recent strains. During the glacial cycles in the Pleistocene, it is believed that the expansion and retraction of forest and the presence of large rivers possibly acted as physical barriers for terrestrial species, promoting divergence by vicariance. Haffer [117] suggested that the Amazon rainforest retracted and expanded during the Pleistocene according to weather events, generating forest refuges, which isolated the founding populations. However, recent evidence suggests that in this period, the Amazon forest remained resilient and therefore afforested [118,119]. Based on these findings, it is possible that marine incursions that generated the formation of three regions contributed to the initial process of diversification of the species of *An*. *oswaldoi* and *An*. *konderi* complexes via allopatric speciation [120]. Subsequently, due to the isolation of the environments and large stretches of rivers in the Pleistocene, the populations of each environments accumulated differences over a long period of time, and when these “populations” later expanded their geographical boundaries, they came into contact with each other (secondary contact areas), already as distinct species. We emphasize that these diversification time estimates in the *An*. *oswaldoi* and *An*. *konderi* complexes may be better supported with the use of more molecular markers and population genomic studies, allowing calibrate the molecular clock.

### Geographical distribution and medical importance

From the molecular data generated in this study and the records of the previous studies, it was possible to elaborate an updated geographic distribution of the Oswaldoi-Konderi complex. Ruiz-Lopez et al. [16] recorded *An*. *oswaldoi* s.s., *An*. *oswaldoi* A and *An*. *konderi* of Sallum in Brazil, while *An*. *oswaldoi* B and *An*. sp. nr. *konderi* were detected in neighboring countries. This is the first study to record the five species of the Oswaldoi-Konderi complex along with the three lineages of *An*. *oswaldoi* A in the Brazilian Amazon region.

*Anopheles oswaldoi* s.s. occurs in the states of Amazonas, Acre and Rondônia (north region) [14,16] and in the states of Espírito Santo, Rio de Janeiro, São Paulo and Paraná (southeast and south regions) [15,16,36]. This disjointed distribution (gap) within Brazil is likely due to the absence of sample collections in the central region of the country, since there is high genetic similarity (99%) among the specimens in the north and southeast regions, including those from the type locality. Previous studies did not report that *An*. *oswaldoi* s.s. is a vector in the southeast region [16]. *Anopheles oswaldoi* A was sampled in Colombia [16] and in Brazil in the states of Amazonas, Acre, Pará, Rondônia and Mato Grosso [7,13,14,16]. In the present study, it was recorded in Coari, Pitinga, Lábrea (Amazonas), Oriximiná (Pará), and Calçoene and Macapá (Amapá). Taken together, these records suggest that *An*. *oswaldoi* A has a large geographic distribution. *Anopheles oswaldoi* B was sampled in Ecuador, Colombia, Trinidad and Tobago, and Venezuela [13,16,56]. In Brazil, it was collected for the first time in the Santana municipality (state of Amapá) [13]. The present study confirms the presence of *An*. *oswaldoi* B in this state, and it was not sympatric with any species. We suspect that this species occurs in all parts of the extreme north of South America, including the Brazilian state of Roraima and along the Guianas Shield (in Guianas and Surinam). Based on previous reports, it has been incriminated as a malaria vector in Colombia [16,54] and Venezuela [56] and as a potential vector in Serra do Navio in Amapá [51]. Recently, Dusfour et al. [57] reported that *An*. *oswaldoi* s.l. was naturally infected with *P*. *falciparum* in French Guyana, which may correspond to *An*. *oswaldoi* B.

*Anopheles konderi* has been reported in the Brazilian states of Acre, Amazonas, Pará, Amapá, and Rondônia, in Perú (Loreto) and in Bolivia (Cochabamba) [16,25]. The role of this species as a malaria vector is largely unknown or it may have been misattributed to *An*. *oswaldoi* s.l. In this study, it was recorded for the first time in Autazes (Amazonas), Oriximiná (Pará), Macapá and Santana Island (Amapá), and Porto Velho and São Miguel (Rondônia). Tadei et al. [42] reported a specimen of *An*. *oswaldoi* s.l. from Cachoeira Porteira (Pará) that was naturally infected with *P*. *falciparum*, a locality situated in the municipality of Oriximiná. In the present study, *An*. *oswaldoi* A and *An*. *konderi* were found to be sympatric in Oriximiná, thereby making us unable to infer the potential vector in that area. *Anopheles* sp. nr. *konderi* was sampled in Colombia, Ecuador and Peru [16], and in Brazil it was collected in the states of Acre and Rondônia by Scarpassa and Conn [14]. In this study, it was recorded in Nova Olinda do Norte (Amazonas) across the Madeira River and co-occurred with *An*. *konderi* in Porto Velho (Rondônia). Although further studies are needed to delimit its geographic distribution, our data suggest that *An*. sp. nr. *konderi* may be widely distributed in Acre.

The present and previous studies discussed above recorded four of the five species in the state of Acre in Brazil (*An*. *oswaldoi* s.s., *An*. *oswaldoi* A, *An*. *konderi* and *An*. sp. nr. *konderi*). Branquinho et al. [49,50] incriminated *An*. *oswaldoi* s.l. as an important malaria vector in Senador Guiomar and Plácido de Castro, in Acre. Therefore, these findings raise doubts that would be the malaria vector in those areas.

Under experimental conditions, Marrelli et al. [58] observed that *An*. *oswaldoi* s.l. may be more susceptible to infection than *An*. *konderi* from Rondônia, whereas Ruiz-Lopez et al. [16] incriminated *An*. *oswaldoi* A, *An*. *oswaldoi* B and *An*. sp. nr. *konderi* based only on their geographic origin. The findings of this study revealed two or more species in the states of Acre, Amazonas, Amapá, Pará and Rondônia, precluding inferences of the malaria vectors in those areas. In fact, the most accurate way to incriminate the members of the Oswaldoi-Konderi complex as potential malaria vectors in transmission areas, especially in areas of co-occurrence, could be the morphological and molecular identification of specimens followed by infection analyses for *Plasmodium* spp. from the head and thorax or, if possible, by dissection of the salivary glands of these mosquitoes. These studies are currently underway in our laboratory.

## Supporting information

S1 Table Comparison between sequences *COI* gene obtained in this study and those available in the GenBank database. (DOC)

S2 Table Variable sites observed for each haplotype in the five species of the Oswaldoi-Konderi complex inferred with the *COI* dataset. (DOC)

S3 Table Information of the haplotypes generated with the *COI* database. (DOC)

S4 Table Mean genetic distances (K2P) inferred from the *COI* dataset after sorting the lineages using the species delimitation bGMYC plugin. (DOC)

S5 Table Variable sites observed for each haplotypes in the five species of the Oswaldoi-Konderi complex inferred with the ITS2 dataset. (DOC)

S6 Table Information of the haplotypes generated with the ITS2 database. (DOC)

S1 Fig Neighbor joining (NJ) tree generated with the *COI* dataset. The values on the branches represent the bootstrapping support calculated with 2,000 replicates.
(TIF)

S2 Fig Maximum parsimony (MP) tree generated with the *COI* dataset. The values on the branches represent the bootstrapping support calculated with 1,000 replicates.
(TIF)

S3 Fig Histogram of the intra (blue color) and interspecific (red color) genetic distances (K2P) obtained with the *COI* dataset. (TIF)

S4 Fig Bayesian Inference (BI) tree generated with all *COI* dataset sequences (the present study + GenBank) based on the HKY + I + G model. **Footnote:** The sequences generated in the present study are shown in **bold**. The sequences downloaded from GenBank are identified by the species name and access number. Red: *An*. *oswaldoi* s.s.; Blue: *An*. *oswaldoi* B; Green: *An*. *oswaldoi* A; Yellow: *An*. *konderi*; Purple: *An*. sp. nr. *konderi*. The yellow symbol (polygon) indicates the sequences of *An*. *konderi* reported by Motoki et al. [25].
(TIF)
