# Supplementary material for: Molecular taxonomy and evolutionary relationships in the Oswaldoi-Konderi complex (Anophelinae: Anopheles: Nyssorhynchus) from the Brazilian Amazon region
Source: PLoS One. 2018 Mar 5;13(3):e0193591. doi: 10.1371/journal.pone.0193591 (PMC5837296; doi:10.1371/journal.pone.0193591)
Supplement: S1 Table — (DOC) [file pone.0193591.s001.doc]

**S1 Table. Comparison between sequences *COI* gene obtained in this study and those available in the GenBank database.**

| **Collected Samples** | | | **SCORE OF IDENTIFY** | **Comparison in the Blastn** | | |
| --- | --- | --- | --- | --- | --- | --- |
| **Nº** | **Code** | **Locality/State** |  | **Author** | **Species** | **Accession Nº** |
| 1 | 474oswssCoa-AM | Coari/AM | 99% | Ruiz-Lopez et al. (2013) | *An. oswaldoi* ss | KF809129 |
| 2 | 475oswssCoa-AM | Coari/AM | 99% | Ruiz-Lopez et al. (2013) | *An. oswaldoi* ss | KF809129 |
| 3 | 478oswssRBra-AC | Rio Branco/AC | 99% | Ruiz-Lopez et al. (2013) | *An. oswaldoi* ss | KF809126 |
| 4 | 482oswssRBra-AC | Rio Branco/AC | 99% | Ruiz-Lopez et al. (2013) | *An. oswaldoi* ss | KF809124 |
| 5 | 470oswssTra-AC | Highway Transacreana /AC | 99% | Ruiz-Lopez et al. (2013) | *An. oswaldoi* ss | KF809126 |
| 6 | 534oswBFGo-AP | Ferreira Gomes/AP | 99% | Rúbio-Palis et al. (2013) | *An. oswaldoi* B | KC555073 |
| 7 | 535oswBFGo-AP | Ferreira Gomes/AP | 99% | Rúbio-Palis et al. (2013) | *An. oswaldoi* B | KC555056 |
| 8 | 541oswBFGo-AP | Ferreira Gomes/AP | 99% | Rúbio-Palis et al. (2013) | *An. oswaldoi* B | KC555056 |
| 9 | 595oswBSNav-AP | Serra do Navio/AP | 98% | Rúbio-Palis et al. (2013) | *An. oswaldoi* B | KC555073 |
| 10 | 596oswBSNav-AP | Serra do Navio/AP | 98% | Rúbio-Palis et al. (2013) | *An. oswaldoi* B | KC555073 |
| 11 | 611oswBSNav-AP | Serra do Navio/AP | 99% | Rúbio-Palis et al. (2013) | *An. oswaldoi* B | KC555056 |
| 12 | 612oswBSNav-AP | Serra do Navio/AP | 98% | Rúbio-Palis et al. (2013) | *An. oswaldoi* B | KC555073 |
| 13 | 543oswBTart-AP | Tartarugalzinho/AP | 98% | Rúbio-Palis et al. (2013) | *An. oswaldoi* B | KC555073 |
| 14 | 461oswACoa-AM | Coari/AM | 100% | Ruiz-Lopez et al. (2013) | *An. oswaldoi* A | KF809050 |
| 15 | 472oswACoa-AM | Coari/AM | 99% | Ruiz-Lopez et al. (2013) | *An. oswaldoi* A | KF809050 |
| 16 | 473oswACoaAM | Coari/AM | 99% | Ruiz-Lopez et al. (2013) | *An. oswaldoi* A | KF809079 |
| 17 | 396oswALab-AM | Lábrea/AM | 99% | Ruiz-Lopez et al. (2013) | *An. oswaldoi* A | KF809068 |
| 18 | 398oswALab-AM | Lábrea/AM | 99% | Ruiz-Lopez et al. (2013) | *An. oswaldoi* A | KF809034 |
| 19 | 400oswALab-AM | Lábrea/AM | 100% | Ruiz-Lopez et al. (2013) | *An. oswaldoi* A | KF809034 |
| 20 | 402oswALab-AM | Lábrea/AM | 100% | Ruiz-Lopez et al. (2013) | *An. oswaldoi* A | KF809079 |
| 21 | 404oswALab-AM | Lábrea/AM | 100% | Ruiz-Lopez et al. (2013) | *An. oswaldoi* A | KF809068 |
| 22 | 406oswALab-AM | Lábrea/AM | 99% | Ruiz-Lopez et al. (2013) | *An. oswaldoi* A | KF809050 |
| 23 | 408oswALab-AM | Lábrea/AM | 99% | Ruiz-Lopez et al. (2013) | *An. oswaldoi* A | KF809079 |
| 24 | 410oswALab-AM | Lábrea/AM | 99% | Ruiz-Lopez et al. (2013) | *An. oswaldoi* A | KF809079 |
| 25 | 412oswALab-AM | Lábrea/AM | 99% | Ruiz-Lopez et al. (2013) | *An. oswaldoi* A | KF809079 |
| 26 | 414oswALab-AM | Lábrea/AM | 99% | Ruiz-Lopez et al. (2013) | *An. oswaldoi* A | KF809079 |
| 27 | 416oswALab-AM | Lábrea/AM | 100% | Ruiz-Lopez et al. (2013) | *An. oswaldoi* A | KF809068 |
| 28 | 418oswALab-AM | Lábrea/AM | 100% | Ruiz-Lopez et al. (2013) | *An. oswaldoi* A | KF809050 |
| 29 | 594oswACalc-AP | Calçoene/AP | 98% | Ruiz-Lopez et al. (2013) | *An. oswaldoi* A | KF809069 |
| 30 | 236oswAMatFo-AP | Mata Fome/AP | 99% | Ruiz-Lopez et al. (2013) | *An. oswaldoi* A | KF809054 |
| 31 | 350oswAPit-AM | Pitinga/AM | 98% | Ruiz-Lopez et al. (2013) | *An. oswaldoi* A | KF809039 |
| 32 | 351oswAPit-AM | Pitinga/AM | 98% | Ruiz-Lopez et al. (2013) | *An. oswaldoi* A | KF809039 |
| 33 | 608oswAPit-AM | Pitinga/AM | 98% | Ruiz-Lopez et al. (2013) | *An. oswaldoi* A | KF809069 |
| 34 | 609oswAPit-AM | Pitinga/AM | 98% | Ruiz-Lopez et al. (2013) | *An. oswaldoi* A | KF809069 |
| 35 | 517oswASCach-PA | Serra do Cachorro/PA | 98% | Ruiz-Lopez et al. (2013) | *An. oswaldoi* A | KF809068 |
| 36 | 522oswASCach-PA | Serra do Cachorro/PA | 98% | Ruiz-Lopez et al. (2013) | *An. oswaldoi* A | KF809034 |
| 37 | 528oswASCach-PA | Serra do Cachorro/PA | 98% | Ruiz-Lopez et al. (2013) | *An. oswaldoi* A | KF809069 |
| 38 | 454oswAStaB-AP | Santa Barbara/AP | 99% | Ruiz-Lopez et al. (2013) | *An. oswaldoi* A | KF809054 |
| 39 | 352kondAut-AM | Autazes/AM | 99% | Sallum et al. (2008) | *An. konderi* | JF923716 |
| 40 | 353kondAut-AM | Autazes/AM | 99% | Sallum et al. (2008) | *An. konderi* | JF923716 |
| 41 | 354kondAut-AM | Autazes/AM | 99% | Sallum et al. (2008) | *An. konderi* | JF923716 |
| 42 | 355kondAut-AM | Autazes/AM | 99% | Sallum et al. (2008) | *An. konderi* | JF923716 |
| 43 | 356kondAut-AM | Autazes/AM | 99% | Sallum et al. (2008) | *An. konderi* | JF923716 |
| 44 | 357kondAut-AM | Autazes/AM | 99% | Sallum et al. (2008) | *An. konderi* | JF923716 |
| 45 | 358kondAut-AM | Autazes/AM | 99% | Sallum et al. (2008) | *An. konderi* | JF923716 |
| 46 | 439kondSMig-RO | São Miguel/RO | 99% | Sallum et al. (2008) | *An. konderi* | JF923716 |
| 47 | 440kondSMig-RO | São Miguel/RO | 99% | Sallum et al. (2008) | *An. konderi* | JF923716 |
| 48 | 441kondSMig-RO | São Miguel/RO | 99% | Sallum et al. (2008) | *An. konderi* | JF923716 |
| 49 | 444kondSMig-RO | São Miguel/RO | 99% | Sallum et al. (2008) | *An. konderi* | JF923716 |
| 50 | 445kondSMig-RO | São Miguel/RO | 99% | Sallum et al. (2008) | *An. konderi* | JF923716 |
| 51 | 447kondSMig-RO | São Miguel/RO | 99% | Sallum et al. (2008) | *An. konderi* | JF923716 |
| 52 | 448kondSMig-RO | São Miguel/RO | 99% | Sallum et al. (2008) | *An. konderi* | JF923716 |
| 53 | 449kondSMig-RO | São Miguel/RO | 99% | Sallum et al. (2008) | *An. konderi* | JF923716 |
| 54 | 450kondSMig-RO | São Miguel/RO | 99% | Sallum et al. (2008) | *An. konderi* | JF923716 |
| 55 | 451kondSMig-RO | São Miguel/RO | 99% | Sallum et al. (2008) | *An. konderi* | JF923716 |
| 56 | 452kondSMig-RO | São Miguel/RO | 99% | Sallum et al. (2008) | *An. konderi* | JF923716 |
| 57 | 497kondAut-AM | Autazes/AM | 99% | Sallum et al. (2008) | *An. konderi* | JF923716 |
| 58 | 498kondAut-AM | Autazes/AM | 99% | Sallum et al. (2008) | *An. konderi* | JF923716 |
| 59 | 367kondAut-AM | Autazes/AM | 99% | Sallum et al. (2008) | *An. konderi* | JF923716 |
| 60 | 368kondAut-AM | Autazes/AM | 99% | Sallum et al. (2008) | *An. konderi* | JF923716 |
| 61 | 597kondSant-AP | Island Santana /AP | 100% | Sallum et al. (2008) | *An. konderi* | JF923716 |
| 62 | 598kondSant-AP | Island Santana /AP | 100% | Sallum et al. (2008) | *An. konderi* | JF923716 |
| 63 | 599kondSant-AP | Island Santana /AP | 100% | Sallum et al. (2008) | *An. konderi* | JF923716 |
| 64 | 584kondMatFo-AP | Mata Fome/AP | 100% | Sallum et al. (2008) | *An. konderi* | JF923716 |
| 65 | 585kondMatFo-AP | Mata Fome/AP | 100% | Sallum et al. (2008) | *An. konderi* | JF923716 |
| 66 | 586kondMatFo-AP | Mata Fome/AP | 100% | Sallum et al. (2008) | *An. konderi* | JF923716 |
| 67 | 488kondPVel-RO | Porto Velho/RO | 99% | Sallum et al. (2008) | *An. konderi* | JF923716 |
| 68 | 499kondSMig-RO | São Miguel/RO | 99% | Sallum et al. (2008) | *An. konderi* | JF923716 |
| 69 | 500kondSMig-RO | São Miguel/RO | 99% | Sallum et al. (2008) | *An. konderi* | JF923716 |
| 70 | 501kondSMig-RO | São Miguel/RO | 99% | Sallum et al. (2008) | *An. konderi* | JF923716 |
| 71 | 502kondSMig-RO | São Miguel/RO | 99% | Sallum et al. (2008) | *An. konderi* | JF923716 |
| 72 | 514kondSCac-PA | Serra do Cachorro/PA | 99% | Sallum et al. (2008) | *An. konderi* | JF923716 |
| 73 | 524kondSCac-PA | Serra do Cachorro/PA | 99% | Sallum et al. (2008) | *An. konderi* | JF923716 |
| 74 | 530kondSCac-PA | Serra do Cachorro/PA | 99% | Sallum et al. (2008) | *An. konderi* | JF923716 |
| 75 | 532kondSCac-PA | Serra do Cachorro/PA | 99% | Sallum et al. (2008) | *An. konderi* | JF923716 |
| 76 | 462kondStB-AP | Santa Barbara/AP | 100% | Sallum et al. (2008) | *An. konderi* | JF923716 |
| 77 | 424nr.kondNON-AM | Nova Olinda do Norte/AM | 99% | Linton et al. (2013) | *An.* sp. nr*. konderi* | KF809138 |
| 78 | 487nrkondPV-RO | Porto Velho/RO | 99% | Linton et al. (2013) | *An.* sp. nr*. konderi* | KF809137 |
| 79 | 477nrkondRBra-AC | Rio Branco/AC | 99% | Linton et al. (2013) | *An.* sp. nr*. konderi* | KF809137 |
| 80 | 479nrkondRBra-AC | Rio Branco/AC | 99% | Linton et al. (2013) | *An.* sp. nr*. konderi* | KF809137 |
| 81 | 481nrkondRBra-AC | Rio Branco/AC | 100% | Linton et al. (2013) | *An.* sp. nr*. konderi* | KF670997 |
| 82 | 471nrkondSMad-AC | Sena Madureira/AC | 100% | Linton et al. (2013) | *An.* sp. nr*. konderi* | KF670997 |
| 83 | 469nrkondTran-AC | Highway Transacreana /AC | 99% | Linton et al. (2013) | *An.* sp. nr*. konderi* | KF670998 |
| 84 | Outgroup | Mata Fome/AP | 99% | Foster et al. (2013) | *Anopheles goeldii* | JF923704 |
| 85 | Outgroup | Mata Fome/AP | 99% | Ruiz-Lopez et al. (2012) | *Anopheles marajoara* | JQ615447 |

**Nº:** Numerical order of the sequences; **Code:** abbreviation of the sequences obtained; **Score of Identify:** Similarity of the sequences obtained in this study with the sequences available in GenBank; **Comparison in Blastn, Autor:** References of the sequences compared; **Species:** Species identified; **Accession Nº:** Accession number of the compared sequences.
