# Supplementary material for: Molecular taxonomy and evolutionary relationships in the Oswaldoi-Konderi complex (Anophelinae: Anopheles: Nyssorhynchus) from the Brazilian Amazon region
Source: PLoS One. 2018 Mar 5;13(3):e0193591. doi: 10.1371/journal.pone.0193591 (PMC5837296; doi:10.1371/journal.pone.0193591)
Supplement: S2 Table — (DOC) [file pone.0193591.s002.doc]

| **H** | **Nº** | **LOCALITY/STATE** |  |  |  |  |  |  |  |  |  |  |  |  |  |  |  | **1** | **1** | **1** | **1** | **1** | **1** | **1** | **2** | **2** | **2** | **2** | **2** | **2** | **2** | **2** | **2** | **2** | **2** | **2** | **2** | **3** | **3** | **3** | **3** | **3** | **3** | **3** | **3** | **3** | **3** |
| --- | --- | --- | --- | --- | --- | --- | --- | --- | --- | --- | --- | --- | --- | --- | --- | --- | --- | --- | --- | --- | --- | --- | --- | --- | --- | --- | --- | --- | --- | --- | --- | --- | --- | --- | --- | --- | --- | --- | --- | --- | --- | --- | --- | --- | --- | --- | --- |
|  |  |  |  | **1** | **1** | **2** | **2** | **3** | **4** | **4** | **5** | **5** | **6** | **7** | **8** | **8** | **9** | **0** | **0** | **2** | **6** | **8** | **8** | **9** | **0** | **0** | **1** | **1** | **2** | **3** | **3** | **5** | **6** | **7** | **7** | **8** | **8** | **1** | **2** | **2** | **3** | **3** | **4** | **4** | **4** | **5** | **6** |
|  |  |  | **3** | **2** | **8** | **4** | **7** | **6** | **2** | **5** | **1** | **2** | **0** | **2** | **1** | **4** | **0** | **5** | **8** | **6** | **8** | **3** | **6** | **5** | **4** | **8** | **6** | **9** | **2** | **1** | **7** | **2** | **8** | **0** | **3** | **5** | **9** | **8** | **1** | **7** | **0** | **9** | **2** | **5** | **8** | **1** | **0** |
| **H01** | 1 | Coari/AM | **A** | **T** | **C** | **A** | **C** | **A** | **A** | **A** | **A** | **T** | **T** | **T** | **T** | **T** | **A** | **T** | **T** | **T** | **A** | **A** | **A** | **C** | **A** | **C** | **A** | **A** | **T** | **A** | **T** | **T** | **T** | **A** | **T** | **T** | **T** | **A** | **T** | **A** | **A** | **T** | **C** | **T** | **T** | **T** | **A** |
| **H02** | 1 | Coari/AM | . | . | . | . | . | . | . | . | . | . | . | . | . | . | . | . | . | . | . | . | . | . | . | . | . | . | . | G | . | . | . | . | . | . | . | . | . | . | . | . | . | . | . | . | . |
| **H03** | 1 | Coari/AM | . | . | . | . | . | . | . | . | . | . | . | . | . | . | . | . | . | . | . | . | . | . | . | . | . | . | . | G | . | . | . | . | . | . | . | . | . | . | . | . | . | . | . | . | . |
| **H04** | 1 | Rio Branco/AC | . | . | . | . | . | . | . | . | . | . | . | . | . | T | . | . | . | . | . | . | . | . | . | . | . | . | . | . | . | . | . | . | . | . | . | . | . | . | . | . | . | . | . | . | . |
| **H05** | 1 | Rio Branco/AC | . | . | . | . | . | . | G | . | . | . | . | . | . | . | . | . | . | . | . | . | . | . | . | . | . | . | . | . | . | . | . | . | . | . | . | . | . | . | . | . | . | . | . | . | . |
| **H06** | 2 | Pitinga/AM | . | C | T | G | T | . | . | . | **T** | **C** | . | C | A | C | . | . | . | . | . | . | . | T | . | T | . | . | C | . | . | C | . | . | . | G | . | . | C | T | . | . | . | C | . | . | . |
| **H07** | 2 | Pitinga/AM and Calçoene/AP | . | C | T | G | T | . | . | . | **T** | **C** | . | C | A | . | . | . | . | . | . | . | . | T | . | T | . | . | C | . | . | C | . | . | . | . | . | . | C | T | . | . | . | C | . | . | . |
| **H08** | 1 | Pitinga/AM | . | C | T | G | T | . | . | . | **T** | **C** | . | C | A | . | . | . | . | T | . | . | . | T | . | T | . | . | C | . | . | C | . | . | . | . | . | . | C | T | . | . | . | C | . | . | . |
| **H09** | 1 | Serra do Cachorro/PA | . | C | T | A | T | . | . | . | **T** | **C** | . | T | A | C | . | . | . | . | . | . | . | T | . | T | . | . | C | . | T | C | . | . | . | . | . | . | C | T | . | . | . | C | . | . | . |
| **H10** | 1 | Serra do Cachorro/PA | . | . | T | . | T | . | . | . | **T** | **C** | . | . | A | . | . | . | . | . | . | . | . | T | . | T | . | . | C | . | T | C | . | . | . | . | C | . | . | T | . | . | . | C | . | . | . |
| **H11** | 2 | Lábrea/AM | . | C | T | G | T | . | . | T | **T** | **C** | . | C | . | . | . | . | . | . | . | . | G | T | . | T | . | . | C | . | C | C | . | . | . | . | . | . | C | . | . | . | T | . | C | . | . |
| **H12** | 1 | Lábrea/AM | . | C | T | G | T | . | . | G | **T** | **C** | . | C | . | . | . | . | . | . | . | . | G | T | . | T | . | . | . | . | C | C | . | . | . | . | . | G | C | . | . | . | T | . | C | . | . |
| **H13** | 1 | Lábrea/AM | . | C | T | G | T | . | . | T | **T** | **C** | . | C | C | . | . | . | . | . | . | . | . | T | . | T | . | . | . | . | C | C | . | . | . | . | . | . | C | . | . | . | T | . | C | . | . |
| **H14** | 1 | Lábrea/AM | . | C | T | G | T | . | . | T | **T** | **C** | . | C | . | . | . | . | . | . | . | . | . | T | . | T | . | . | . | . | C | C | . | . | . | . | . | . | C | . | . | . | T | . | C | . | . |
| **H15** | 1 | Lábrea/AM | . | C | T | G | T | . | . | T | **T** | **C** | . | C | . | . | . | . | . | . | . | . | G | T | . | T | . | . | . | . | C | C | . | . | . | . | . | . | C | . | . | . | T | . | C | . | . |
| **H16** | 2 | Lábrea/AM | . | C | T | G | T | . | . | G | **T** | **C** | . | C | . | . | . | . | . | . | . | . | G | T | . | T | . | . | . | . | C | C | . | . | . | . | . | . | C | . | . | . | T | . | C | . | . |
| **H17** | 1 | Lábrea/AM | . | C | T | G | T | . | . | G | **T** | **C** | . | C | . | . | . | . | . | . | . | . | G | T | . | T | . | . | . | . | C | C | . | . | . | . | . | . | C | . | . | . | T | . | C | . | . |
| **H18** | 1 | Lábrea/AM | . | C | T | G | T | . | . | T | **T** | **C** | . | C | . | . | . | . | . | . | . | . | G | T | . | T | . | . | . | . | C | C | . | . | . | . | . | . | C | . | . | . | T | . | C | . | . |
| **H19** | 1 | Lábrea/AM | . | C | T | G | T | . | . | T | **T** | **C** | . | C | . | . | . | . | . | . | . | . | G | T | . | T | . | . | . | . | C | C | . | . | . | . | . | . | C | . | . | . | T | . | C | . | . |
| **H20** | 2 | Lábrea and Coari/AM | . | C | T | G | T | . | . | G | **T** | **C** | . | C | . | . | . | . | . | . | . | . | G | T | . | T | . | . | . | . | C | C | . | . | . | . | . | . | C | . | . | . | T | . | C | . | . |
| **H21** | 1 | Coari/AM | . | C | T | G | T | . | . | G | **T** | **C** | . | C | . | . | . | . | . | . | . | . | G | T | . | T | . | . | . | . | C | C | . | . | . | . | . | . | C | . | . | . | T | . | C | . | . |
| **H22** | 1 | Coari/AM | . | C | T | G | T | . | . | T | **T** | **C** | . | C | . | . | . | . | . | . | . | . | G | T | . | T | . | . | . | . | C | C | . | . | . | . | . | . | C | . | . | . | T | . | C | . | . |
| **H23** | 1 | Mata Fome/AP | . | C | T | . | T | . | . | G | **T** | **C** | C | C | . | . | . | . | . | . | . | . | . | T | . | T | . | G | . | . | C | C | . | . | . | . | . | . | C | . | . | . | T | C | C | . | . |
| **H24** | 1 | Santa Barbara/AP | . | C | T | . | T | . | . | G | **T** | **C** | C | C | . | . | . | . | . | . | G | . | . | T | . | T | . | G | . | . | C | C | . | . | . | . | . | . | C | . | . | . | T | C | C | . | . |
| **H25** | 1 | Serra do Cachorro/PA | . | C | T | G | T | . | . | . | **T** | **C** | . | . | T | T | . | . | . | . | . | . | . | T | . | T | . | . | T | . | C | C | . | . | . | . | T | . | C | A | . | . | T | C | . | . | . |
| **H26** | 1 | Ferreira Gomes/AP | . | . | T | . | . | . | . | . | . | . | . | . | G | . | T | C | . | . | . | G | G | T | . | . | . | . | . | . | C | . | **C** | . | . | . | . | G | . | . | . | . | T | . | . | . | . |
| **H27** | 1 | Serra do Navio/AP | A | T | C | . | . | . | . | . | . | . | . | . | G | . | T | C | . | . | . | . | G | T | . | . | . | . | . | . | C | . | **C** | . | . | . | . | . | . | . | . | . | . | . | . | C | . |
| **H28** | 1 | Ferreira Gomes/AP | . | . | . | . | . | . | . | . | . | . | . | . | G | . | T | C | . | . | . | G | G | T | . | . | . | . | . | . | C | . | **C** | . | . | . | . | G | . | . | . | . | T | . | . | . | . |
| **H29** | 4 | Ferreira Gomes and Serra do Navio/AP | . | . | . | . | . | . | G | G | . | . | . | . | A | . | . | . | . | . | . | . | G | T | . | . | . | . | . | . | C | C | **C** | . | . | . | . | . | . | . | . | . | T | C | . | . | . |
| **H30** | 1 | Tartarugalzinho/AP | G | . | . | . | . | . | G | G | . | . | . | . | A | . | . | . | . | . | . | . | G | T | . | . | . | . | . | . | C | C | **C** | . | . | . | . | . | . | . | . | . | T | C | . | . | . |
| **H31** | 7 | Santa Barbara, Island Santana and Mata fome/AP | . | . | T | . | T | . | . | . | G | . | . | . | C | C | . | . | . | C | . | . | . | T | **G** | T | . | . | . | . | C | . | . | G | . | . | C | . | . | . | G | . | . | C | . | . | . |
| **H32** | 3 | Autazes/AM | . | . | T | . | T | G | . | . | G | . | . | . | C | C | . | . | . | C | . | . | . | T | **G** | T | . | . | . | . | C | . | . | G | . | . | C | . | . | . | . | . | . | C | . | . | . |
| **H33** | 3 | Autazes/AM | . | . | T | . | T | . | . | . | G | . | . | . | C | C | . | . | C | C | . | . | . | T | **G** | T | . | . | . | . | C | . | . | G | . | . | C | . | . | . | G | . | . | C | . | . | . |
| **H34** | 3 | Autazes/AM | . | . | T | . | T | G | . | . | G | . | . | . | C | C | G | . | . | C | . | . | . | T | **G** | T | . | . | . | . | C | . | . | G | . | . | C | . | . | . | G | . | . | C | . | . | . |
| **H35** | 2 | Autazes/AM | . | . | T | . | T | . | . | . | . | . | . | . | C | C | . | . | . | C | . | . | . | T | **G** | T | . | . | . | . | C | . | . | G | C | . | C | . | . | . | G | . | . | C | . | . | . |
| **H36** | 17 | São Miguel/RO and Serra do Cachorro/PA | . | . | T | . | T | . | . | . | G | . | . | . | C | C | . | . | . | C | . | . | . | T | **G** | T | G | . | . | . | C | . | . | . | . | . | C | . | . | . | G | . | . | C | . | . | . |
| **H37** | 2 | São Miguel and Porto Velho/RO | A | . | T | . | T | . | . | . | G | . | . | . | C | C | . | . | . | C | . | . | . | T | **G** | T | G | . | . | . | C | . | . | . | . | . | C | . | . | . | G | . | . | C | . | . | G |
| **H38** | 1 | Serra do Cachorro/PA | . | . | T | . | T | . | . | . | G | . | . | . | C | C | . | . | . | . | . | . | . | T | **G** | T | . | . | . | . | C | . | . | . | . | . | C | . | . | . | G | . | . | C | . | . | . |
| **H39** | 2 | Rio Branco/AC | . | . | T | . | T | . | . | . | . | . | . | . | C | C | . | . | . | . | . | . | . | T | . | T | . | . | . | . | C | C | . | . | C | . | C | G | . | . | . | . | T | . | . | . | . |
| **H40** | 1 | Porto Velho/RO | . | . | T | . | T | . | . | . | . | . | . | . | C | C | . | . | . | . | . | . | . | T | . | T | . | . | . | . | C | C | . | G | C | . | C | G | . | . | . | . | T | . | . | . | . |
| **H41** | 1 | Nova Olinda do Norte/AM | . | . | T | . | T | . | . | . | . | . | . | . | . | C | . | . | . | . | . | . | . | T | . | T | . | . | C | . | C | C | . | . | C | . | C | G | . | . | . | A | T | . | . | . | . |
| **H42** | 1 | Highway Transacreana/AC | . | . | T | . | T | . | . | . | . | . | . | . | . | C | . | . | . | . | . | G | . | T | . | T | . | . | C | . | C | . | . | G | . | . | C | G | . | . | . | . | T | . | . | . | . |
| **H43** | 2 | Rio Branco and Sena Madureira/AC | . | . | T | . | T | . | . | . | . | . | . | . | . | C | . | . | . | . | . | G | . | T | . | T | . | . | C | . | C | . | . | G | C | . | C | G | . | . | . | . | T | C | . | . | . |

**S2 Table. Variable sites observed for each haplotype in the five species of the Oswaldoi-Konderi complex inferred with the *COI* dataset.**

Continue

| **H** | **Nº** | **LOCALITY/STATE** | 3 | 3 | 3 | 3 | 4 | 4 | 4 | 4 | 4 | 4 | 4 | 4 | 4 | 4 | 4 | 4 | 4 | 4 | 5 | 5 | 5 | 5 | 5 | 5 | 5 | 5 | 5 | 5 | 5 | 5 | 5 | 5 | 5 | 6 | 6 | 6 | 6 | 6 | 6 | 6 | 6 | 6 | 6 | 6 | 6 |
| --- | --- | --- | --- | --- | --- | --- | --- | --- | --- | --- | --- | --- | --- | --- | --- | --- | --- | --- | --- | --- | --- | --- | --- | --- | --- | --- | --- | --- | --- | --- | --- | --- | --- | --- | --- | --- | --- | --- | --- | --- | --- | --- | --- | --- | --- | --- | --- |
|  |  |  | 6 | 7 | 7 | 8 | 0 | 0 | 0 | 1 | 3 | 3 | 5 | 5 | 6 | 7 | 7 | 8 | 8 | 9 | 0 | 0 | 1 | 2 | 3 | 4 | 5 | 5 | 6 | 6 | 7 | 7 | 8 | 8 | 9 | 0 | 0 | 1 | 1 | 1 | 2 | 2 | 3 | 3 | 3 | 4 | 5 |
|  |  |  | 6 | 2 | 5 | 4 | 2 | 5 | 8 | 7 | 0 | 8 | 3 | 9 | 5 | 1 | 7 | 0 | 7 | 4 | 1 | 4 | 0 | 8 | 1 | 4 | 5 | 9 | 1 | 7 | 0 | 6 | 0 | 5 | 1 | 3 | 9 | 2 | 5 | 8 | 1 | 4 | 0 | 3 | 6 | 8 | 4 |
| **H01** | 1 | Coari/AM | **T** | **T** | **A** | **T** | **T** | **C** | **T** | **A** | **T** | **T** | **T** | **T** | **T** | **G** | **G** | **A** | **T** | **G** | **T** | **A** | **T** | **A** | **T** | **T** | **T** | **T** | **G** | **A** | **T** | **T** | **T** | **A** | **C** | **T** | **A** | **T** | **T** | **C** | **T** | **T** | **A** | **A** | **C** | **T** | **T** |
| **H02** | 1 | Coari/AM | . | . | G | . | . | . | . | . | . | . | . | . | . | A | A | . | . | . | . | . | . | . | . | . | . | . | . | . | . | . | . | . | . | . | . | C | . | . | . | . | . | . | T | C | . |
| **H03** | 1 | Coari/AM | . | . | G | . | . | . | . | . | . | . | . | . | . | A | A | . | . | . | . | . | . | . | . | . | . | . | . | . | . | . | . | . | . | . | . | C | . | . | . | . | . | . | . | C | . |
| **H04** | 1 | Rio Branco/AC | . | . | . | . | . | . | . | . | . | . | . | . | . | A | A | . | . | . | . | . | . | . | . | . | . | . | . | . | . | . | . | . | . | . | . | C | . | . | . | . | . | . | . | . | . |
| **H05** | 1 | Rio Branco/AC | . | . | . | . | . | . | . | . | . | . | . | . | . | A | A | . | . | A | . | . | . | . | . | . | . | . | . | . | . | . | . | . | . | . | . | C | . | T | . | . | . | . | . | . | . |
| **H06** | 2 | Pitinga/AM | . | . | . | . | C | T | . | G | . | **C** | . | . | C | A | A | . | . | . | . | . | . | . | . | . | . | . | A | G | C | . | . | . | T | . | . | . | . | . | . | A | T | . | T | C | C |
| **H07** | 2 | Pitinga/AM and Calçoene/AP | . | . | . | . | C | T | . | G | . | **C** | . | . | C | A | A | . | . | . | . | . | . | . | . | . | . | . | A | G | C | . | . | . | T | . | . | . | . | . | . | A | T | . | . | C | C |
| **H08** | 1 | Pitinga/AM | . | . | . | . | C | T | . | G | . | **C** | . | . | C | A | A | . | . | . | . | . | . | . | . | . | . | . | A | G | C | . | . | . | T | . | . | . | . | . | . | A | T | . | T | C | C |
| **H09** | 1 | Serra do Cachorro/PA | . | . | G | . | C | T | . | G | . | **C** | . | . | C | A | A | . | . | . | . | . | T | . | . | . | T | . | A | G | C | . | . | . | T | . | . | . | . | . | . | A | G | . | T | C | . |
| **H10** | 1 | Serra do Cachorro/PA | . | . | . | . | C | T | . | . | . | **C** | . | . | C | A | A | . | . | . | . | . | A | . | . | . | . | . | A | . | C | . | . | . | T | . | . | . | T | T | . | . | . | . | . | C | . |
| **H11** | 2 | Lábrea/AM | . | . | G | C | C | T | . | G | . | **C** | . | . | . | A | A | . | . | . | . | . | . | . | . | . | . | . | A | G | C | . | . | . | T | . | G | . | . | . | C | A | T | . | T | C | C |
| **H12** | 1 | Lábrea/AM | . | . | . | C | C | T | . | G | . | **C** | . | . | . | A | A | . | . | . | . | . | . | . | . | . | . | . | A | G | C | . | . | . | T | . | G | . | . | . | C | A | T | . | T | C | C |
| **H13** | 1 | Lábrea/AM | . | . | G | C | C | T | . | G | . | **C** | . | . | . | A | A | . | . | . | . | . | . | . | . | . | . | . | A | G | C | . | . | . | T | . | G | . | . | . | C | A | T | . | T | C | C |
| **H14** | 1 | Lábrea/AM | . | . | G | C | C | T | . | G | . | **C** | . | . | . | A | A | . | . | . | . | . | . | . | . | . | . | . | A | G | C | . | . | . | T | . | G | . | T | . | C | A | T | . | T | C | C |
| **H15** | 1 | Lábrea/AM | . | . | G | C | C | T | . | G | . | **C** | . | . | . | A | A | . | . | . | . | . | . | . | . | . | . | . | A | G | C | . | . | . | T | . | G | . | . | . | C | A | T | . | T | C | C |
| **H16** | 2 | Lábrea/AM | . | . | . | C | C | T | . | G | . | **C** | . | . | . | A | A | . | . | . | . | . | . | . | . | . | . | . | A | G | C | . | . | . | T | . | G | . | . | . | C | A | T | . | T | C | C |
| **H17** | 1 | Lábrea/AM | . | . | G | C | C | T | . | G | . | **C** | . | . | . | A | A | . | . | . | . | . | . | . | . | . | . | . | A | G | C | . | . | . | T | . | G | . | . | T | C | A | T | . | T | C | C |
| **H18** | 1 | Lábrea/AM | . | . | G | C | C | T | . | G | . | **C** | . | . | . | A | A | . | . | . | . | . | . | . | . | . | . | . | A | G | C | . | . | . | T | . | G | . | . | T | C | A | T | . | T | C | C |
| **H19** | 1 | Lábrea/AM | . | . | G | C | C | T | . | G | . | **C** | . | . | . | A | A | . | . | . | . | . | . | . | . | . | . | . | A | G | C | . | . | . | T | . | G | . | . | . | . | A | T | . | T | C | C |
| **H20** | 2 | Lábrea and Coari/AM | . | . | . | C | C | T | . | G | . | **C** | . | . | . | A | A | . | . | . | . | . | . | . | . | . | . | . | A | G | C | . | . | . | T | . | G | . | . | . | C | A | T | . | T | C | C |
| **H21** | 1 | Coari/AM | . | . | G | C | C | T | . | G | . | **C** | . | . | . | A | A | . | . | . | . | . | . | . | . | . | . | . | A | G | C | . | . | . | T | . | G | . | . | . | . | A | T | . | T | C | C |
| **H22** | 1 | Coari/AM | . | . | G | C | C | T | . | G | . | **C** | . | . | . | A | A | . | . | . | . | . | . | . | . | . | . | . | A | G | . | . | . | . | T | . | G | . | . | . | C | A | T | . | T | C | C |
| **H23** | 1 | Mata Fome/AP | . | . | . | C | C | T | . | G | . | **C** | . | . | . | A | A | . | . | . | . | . | . | . | . | . | . | . | A | G | C | . | . | . | T | . | . | . | . | . | . | A | T | . | T | . | C |
| **H24** | 1 | Santa Barbara/AP | . | . | . | C | C | T | . | G | . | **C** | . | . | . | A | A | . | . | . | . | . | . | . | . | . | . | . | A | G | C | . | . | . | T | . | . | . | . | . | . | A | T | . | T | . | C |
| **H25** | 1 | Serra do Cachorro/PA | . | . | . | . | C | T | . | G | . | **C** | T | . | C | A | A | . | . | . | . | . | T | . | . | . | T | . | A | . | C | . | . | . | T | . | . | . | . | C | . | A | T | . | T | C | . |
| **H26** | 1 | Ferreira Gomes/AP | **C** | **C** | . | . | . | T | . | G | . | . | . | . | . | A | A | **G** | C | . | A | . | . | . | . | **C** | . | . | A | . | . | . | . | . | . | . | **T** | C | **.** | T | . | C | . | . | T | . | . |
| **H27** | 1 | Serra do Navio/AP | **C** | **C** | G | . | . | T | . | G | . | . | . | . | . | . | A | **G** | C | . | A | . | . | . | . | **C** | . | C | A | . | . | . | . | . | . | . | **T** | C | **.** | T | . | C | . | . | T | . | . |
| **H28** | 1 | Ferreira Gomes/AP | **C** | **C** | . | . | . | T | . | G | . | . | . | . | . | A | A | **G** | C | . | A | . | . | . | . | **C** | . | . | A | . | . | . | . | . | . | . | **T** | C | **.** | T | . | C | . | . | . | . | . |
| **H29** | 4 | Ferreira Gomes and Serra do Navio/AP | **C** | **C** | . | . | . | T | . | G | . | . | . | . | . | A | A | **G** | C | . | A | . | . | . | . | **C** | . | C | A | . | . | . | . | . | . | C | **T** | C | **.** | T | . | . | . | . | . | . | . |
| **H30** | 1 | Tartarugalzinho/AP | **C** | **C** | . | . | . | T | . | G | . | . | . | . | . | A | A | **G** | C | . | A | . | . | . | . | **C** | . | C | A | . | . | . | . | . | . | C | **T** | C | **.** | T | . | . | . | . | . | . | . |
| **H31** | 7 | Santa Barbara, Island Santana and Mata fome/AP | . | . | . | . | . | T | . | . | . | . | . | . | . | A | A | . | C | . | . | . | . | . | C | . | . | . | A | . | . | . | **C** | . | T | . | . | . | . | . | . | . | . | . | . | . | C |
| **H32** | 3 | Autazes/AM | . | . | . | . | . | T | . | . | . | . | . | . | . | A | A | . | C | . | . | . | . | . | C | . | G | . | A | . | . | . | **C** | . | T | . | . | . | . | . | . | . | . | . | . | . | C |
| **H33** | 3 | Autazes/AM | . | . | . | . | . | T | . | . | . | . | . | . | . | A | A | . | C | . | . | . | . | . | C | . | . | . | A | . | . | . | **C** | . | T | . | . | . | . | . | . | . | . | G | . | . | C |
| **H34** | 3 | Autazes/AM | . | . | . | . | . | T | . | . | . | . | . | . | . | A | A | . | C | . | . | . | . | . | C | . | . | . | A | . | . | . | **C** | . | T | . | . | . | . | . | . | . | . | . | . | . | C |
| **H35** | 2 | Autazes/AM | . | . | . | . | . | T | . | . | . | . | . | . | . | A | A | . | C | . | . | . | . | . | C | . | . | . | A | . | . | . | **C** | . | T | . | . | . | . | . | . | . | . | . | . | . | C |
| **H36** | 17 | São Miguel/RO and Serra do Cachorro/PA | . | . | . | . | . | T | . | . | . | . | . | . | . | A | A | . | . | . | . | . | . | . | C | . | . | . | A | . | . | . | **C** | . | T | . | . | . | . | . | . | . | . | . | . | . | C |
| **H37** | 2 | São Miguel and Porto Velho/RO | . | . | . | . | . | T | . | . | . | . | . | . | . | A | A | . | . | . | . | . | . | . | C | . | . | . | A | . | . | . | **C** | . | T | C | . | . | . | . | . | . | . | . | . | . | C |
| **H38** | 1 | Serra do Cachorro/PA | . | . | . | . | . | T | . | . | . | . | . | . | . | A | A | . | . | . | . | . | . | . | C | . | . | . | A | . | . | . | **C** | . | T | . | . | . | . | . | . | . | . | . | . | . | C |
| **H39** | 2 | Rio Branco/AC | . | . | **T** | . | C | T | . | . | C | . | . | . | . | A | A | . | . | . | A | **G** | . | . | C | . | . | . | A | . | . | **A** | . | . | T | . | . | . | **C** | T | . | . | . | G | T | C | C |
| **H40** | 1 | Porto Velho/RO | . | . | **T** | . | C | T | . | . | C | . | . | . | . | A | A | . | . | . | A | **G** | . | C | C | . | . | . | A | . | . | **A** | . | . | T | . | . | . | **C** | . | . | . | . | . | T | C | C |
| **H41** | 1 | Nova Olinda do Norte/AM | . | . | **T** | . | C | T | C | . | . | . | . | . | . | A | A | . | . | . | A | **G** | . | . | C | . | . | . | A | . | . | **A** | . | G | T | . | . | . | **C** | T | . | . | . | . | T | C | C |
| **H42** | 1 | Highway Transacreana/AC | . | . | **T** | . | . | T | . | . | C | . | C | C | C | A | A | . | . | . | A | **G** | . | . | C | . | . | . | A | G | . | **A** | . | . | T | . | . | . | **C** | T | . | . | . | G | . | C | C |
| **H43** | 2 | Rio Branco and Sena Madureira/AC | . | . | **T** | . | . | T | . | . | C | . | C | . | C | A | A | . | . | . | A | **G** | . | . | C | . | . | . | A | G | . | **A** | . | . | T | . | . | . | **C** | T | . | . | . | G | . | C | C |

H: haplotypes; Nº: Individuals number observed in each haplotype; AC: Acre; AM: Amazonas; AP: Amapá, PA: Pará; RO: Rondônia. The fixed sites between species are highlighted in gray color.
