# Supplementary material for: Molecular taxonomy and evolutionary relationships in the Oswaldoi-Konderi complex (Anophelinae: Anopheles: Nyssorhynchus) from the Brazilian Amazon region
Source: PLoS One. 2018 Mar 5;13(3):e0193591. doi: 10.1371/journal.pone.0193591 (PMC5837296; doi:10.1371/journal.pone.0193591)
Supplement: S3 Table — (DOC) [file pone.0193591.s003.doc]

**S3 Table.** **Information of the haplotypes generated with the *COI* database.**

| **H** | **Nº** | **SPECIES** | **LOCALITIY** |
| --- | --- | --- | --- |
| **H1** | 1 | *A. oswaldoi* s.s. | Highway Transacreana |
| **H2** | 1 | *A. oswaldoi* s.s. | Coari |
| **H3** | 1 | *A. oswaldoi* s.s. | Coari |
| **H4** | 1 | *A. oswaldoi* s.s. | Rio Branco |
| **H5** | 1 | *A. oswaldoi* s.s. | Rio Branco |
| **H6** | 2 | *A. oswaldoi* A | Pitinga |
| **H7** | 2 | *A. oswaldoi* A | Pitinga and Calçoene |
| **H8** | 1 | *A. oswaldoi* A | Pitinga |
| **H9** | 1 | *A. oswaldoi* A | Serra do Cachorro |
| **H10** | 1 | *A. oswaldoi* A | Serra do Cachorro |
| **H11** | 2 | *A. oswaldoi* A | Lábrea |
| **H12** | 1 | *A. oswaldoi* A | Lábrea |
| **H13** | 1 | *A. oswaldoi* A | Lábrea |
| **H14** | 1 | *A. oswaldoi* A | Lábrea |
| **H15** | 1 | *A. oswaldoi* A | Lábrea |
| **H16** | 2 | *A. oswaldoi* A | Lábrea |
| **H17** | 1 | *A. oswaldoi* A | Lábrea |
| **H18** | 1 | *A. oswaldoi* A | Lábrea |
| **H19** | 1 | *A. oswaldoi* A | Lábrea |
| **H20** | 2 | *A. oswaldoi* A | Lábrea and Coari |
| **H21** | 1 | *A. oswaldoi* A | Coari |
| **H22** | 1 | *A. oswaldoi* A | Coari |
| **H23** | 1 | *A. oswaldoi* A | Mata Fome |
| **H24** | 1 | *A. oswaldoi* A | Santa Barbara |
| **H25** | 1 | *A. oswaldoi* A | Serra do Cachorro |
| **H26** | 4 | *A. oswaldoi* B | Ferreira Gomes (1) and Serra do Navio (3) |
| **H27** | 1 | *A. oswaldoi* B | Tartarugalzinho |
| **H28** | 1 | *A. oswaldoi* B | Ferreira Gomes |
| **H29** | 1 | *A. oswaldoi* B | Serra do Navio |
| **H30** | 1 | *A. oswaldoi* B | Ferreira Gomes |
| **H31** | 7 | *A. konderi* | Santa Barbara (1); Santana Island (3) and Mata Fome (3) |
| **H32** | 3 | *A. konderi* | Autazes |
| **H33** | 3 | *A. konderi* | Autazes |
| **H34** | 3 | *A. konderi* | Autazes |
| **H35** | 2 | *A. konderi* | Autazes |
| **H36** | 17 | *A. konderi* | São Miguel (14) and Serra do Cachorro (3) |
| **H37** | 2 | *A. konderi* | São Miguel and Porto Velho |
| **H38** | 1 | *A. konderi* | Serra do Cachorro |
| **H39** | 2 | *A.* sp. nr. *konderi* | Rio Branco |
| **H40** | 1 | *A.* sp. nr. *konderi* | Porto Velho |
| **H41** | 1 | *A.* sp. nr. *konderi* | Nova Olinda do Norte |
| **H42** | 1 | *A.* sp. nr. *konderi* | Highway Transacreana |
| **H43** | 2 | *A.* sp. nr. *konderi* | Sena Madureira and Rio Branco |

H: Haplotyes; N°: Absolute frequency of individuals observed in each haplotype. Within parentheses are the numbers of individuals observed for each haplotypes in each locality.
