# Supplementary material for: Molecular taxonomy and evolutionary relationships in the Oswaldoi-Konderi complex (Anophelinae: Anopheles: Nyssorhynchus) from the Brazilian Amazon region
Source: PLoS One. 2018 Mar 5;13(3):e0193591. doi: 10.1371/journal.pone.0193591 (PMC5837296; doi:10.1371/journal.pone.0193591)
Supplement: S4 Table — (DOC) [file pone.0193591.s004.doc]

**S4 Table. Mean genetic distances (K2P) inferred from the *COI* dataset after sorting the lineages using the species delimitation bGMYC plugin.**

| **LINEAGES** | **oswss** | **oswA1** | **oswA2** | **oswA3** | **oswB1** | **oswB2** | **kond1** | **kond2** | **nr1** | **nr2** |
| --- | --- | --- | --- | --- | --- | --- | --- | --- | --- | --- |
| ***osw*ss** | **0.007** |  |  |  |  |  |  |  |  |  |
| ***osw*A1** | 0.048 | **0.004** |  |  |  |  |  |  |  |  |
| ***osw*A2** | 0.054 | 0.024 | **0.003** |  |  |  |  |  |  |  |
| ***osw*A3** | 0.053 | 0.023 | 0.015 | **0.002** |  |  |  |  |  |  |
| ***osw*B1** | 0.039 | 0.062 | 0.061 | 0.058 | **0.001** |  |  |  |  |  |
| ***osw*B2** | 0.039 | 0.067 | 0.062 | 0.065 | 0.021 | **0.009** |  |  |  |  |
| ***kon*1** | 0.039 | 0.047 | 0.056 | 0.049 | 0.052 | 0.054 | **0.004** |  |  |  |
| ***kon*2** | 0.037 | 0.044 | 0.054 | 0.047 | 0.053 | 0.055 | 0.006 | **0.001** |  |  |
| **nr1** | 0.046 | 0.050 | 0.050 | 0.052 | 0.057 | 0.055 | 0.038 | 0.036 | **0.007** |  |
| **nr2** | 0.051 | 0.053 | 0.060 | 0.060 | 0.063 | 0.060 | 0.039 | 0.040 | 0.019 | **0.003** |

The significant values of divergence obtained between lineages are underlined. The **bold** values are intra lineages. **oswss:** *An. oswaldoi* s.s., **oswA:** *An. oswaldoi* A, **oswB:** *An. oswaldoi* B, **kond:** *An. konderi*, **nr.kond:** *An.* sp. nr. *konderi.*
