# Supplementary material for: Molecular taxonomy and evolutionary relationships in the Oswaldoi-Konderi complex (Anophelinae: Anopheles: Nyssorhynchus) from the Brazilian Amazon region
Source: PLoS One. 2018 Mar 5;13(3):e0193591. doi: 10.1371/journal.pone.0193591 (PMC5837296; doi:10.1371/journal.pone.0193591)
Supplement: S5 Table — (DOC) [file pone.0193591.s005.doc]

**S5 Table.** **Variable sites observed for each haplotypes in the five species of the Oswaldoi-Konderi complex inferred with the ITS2 dataset.**

| **H** | **SPECIES** | **Nº** | **1** | **2** | **2** | **3** | **3** | **3** | **3** | **3** | **3** | **3** | **3** | **4** | **4** | **4** | **4** | **4** | **4** | **4** | **4** | **4** |
| --- | --- | --- | --- | --- | --- | --- | --- | --- | --- | --- | --- | --- | --- | --- | --- | --- | --- | --- | --- | --- | --- | --- |
|  |  |  | **9** | **4** | **9** | **1** | **2** | **2** | **5** | **7** | **8** | **9** | **9** | **3** | **4** | **4** | **5** | **5** | **6** | **7** | **7** | **9** |
|  |  |  | **4** | **6** | **7** | **9** | **5** | **9** | **1** | **6** | **9** | **3** | **7** | **2** | **0** | **6** | **1** | **3** | **2** | **1** | **9** | **7** |
| H1 | *An. oswaldoi s.s.* | **1** | A | C | T | C | - | A | C | A | C | **C** | A | **T** | C | C | C | G | G | - | - | - |
| H2 | *An. oswaldoi s.s.* | **2** | A | C | T | C | - | A | C | A | C | **C** | A | **T** | C | G | T | G | G | A | C | - |
| H3 | *An. oswaldo*i A | **9** | **G** | C | T | C | - | A | C | A | C | A | A | C | C | A | T | G | G | G | A | - |
| H4 | *An. oswaldo*i A | **1** | **G** | C | T | A | - | A | C | A | C | A | A | C | C | A | T | G | G | G | A | - |
| H5 | *An. oswaldoi* B | **5** | A | T | T | C | A | A | **-** | A | **A** | **G** | **C** | C | C | A | T | **C** | **A** | A | C | A |
| H6 | *An. konderi* | **2** | A | T | A | A | A | G | C | A | C | A | A | C | **T** | A | T | G | G | G | A | A |
| H7 | *An. konderi* | **3** | A | T | T | C | A | A | C | A | C | A | A | C | **T** | A | T | G | G | G | A | G |
| H8 | *An*. sp. nr. *konderi* | **1** | A | T | T | A | **G** | G | C | A | C | A | A | C | C | A | T | G | G | G | A | - |
| H9 | *An*. sp. nr. *konderi* | **2** | A | T | A | A | **G** | G | C | A | C | A | A | C | C | A | T | G | G | A | C | A |
| H10 | *An*. sp. nr. *konderi* | **1** | A | T | A | A | **G** | G | C | T | C | A | A | C | C | A | T | G | G | A | C | - |

H: Haplotypes; Nº: Number of individuals in each haplotype; AC: Acre; AM: Amazonas; AP: Amapá, PA: Pará; RO: Rondônia. The fixed sites between species are highlight in gray color; -: deletions.
