# Supplementary material for: Molecular taxonomy and evolutionary relationships in the Oswaldoi-Konderi complex (Anophelinae: Anopheles: Nyssorhynchus) from the Brazilian Amazon region
Source: PLoS One. 2018 Mar 5;13(3):e0193591. doi: 10.1371/journal.pone.0193591 (PMC5837296; doi:10.1371/journal.pone.0193591)
Supplement: S6 Table — (DOC) [file pone.0193591.s006.doc]

**S6 Table. Information of the haplotypes generated with the ITS2 database.**

| **H** | **Nº** | **SPECIES** | **LOCALITY** |
| --- | --- | --- | --- |
| H1 | 1 | *An. oswaldoi* s.s. | Coari |
| H2 | 2 | *An. oswaldoi* s.s. | Coari and Rio Branco |
| H3 | 9 | *An. oswaldoi* A | Pitinga (3), Coari (1), Serra do Cachorro (4) and Calçoene (1) |
| H4 | 1 | *An. oswaldoi* A | Mata Fome |
| H5 | 5 | *An. oswaldoi* B | Serra do Navio (2), Ferreira Gomes (2) and Tartarugalzinho (1) |
| H6 | 2 | *An. konderi* | Santa Barbara and Mata Fome |
| H7 | 3 | *An. konderi* | Mata Fome (2) and São Miguel (1) |
| H8 | 1 | *An.* sp. nr. *konderi* | Nova Olinda do Norte |
| H9 | 2 | *An.* sp. nr. *konderi* | Sena Madureira and Highway Transacreana |
| H10 | 1 | *An.* sp. nr. *konderi* | Rio Branco |

H: Haplotyes; N°: Absolute frequency of individuals observed in each haplotype. Within parentheses are the numbers of individuals observed for each haplotypes in each locality.
