# Supplementary figures and images for: Molecular taxonomy and evolutionary relationships in the Oswaldoi-Konderi complex (Anophelinae: Anopheles: Nyssorhynchus) from the Brazilian Amazon region
Source: PLoS One. 2018 Mar 5;13(3):e0193591. doi: 10.1371/journal.pone.0193591 (PMC5837296; doi:10.1371/journal.pone.0193591)

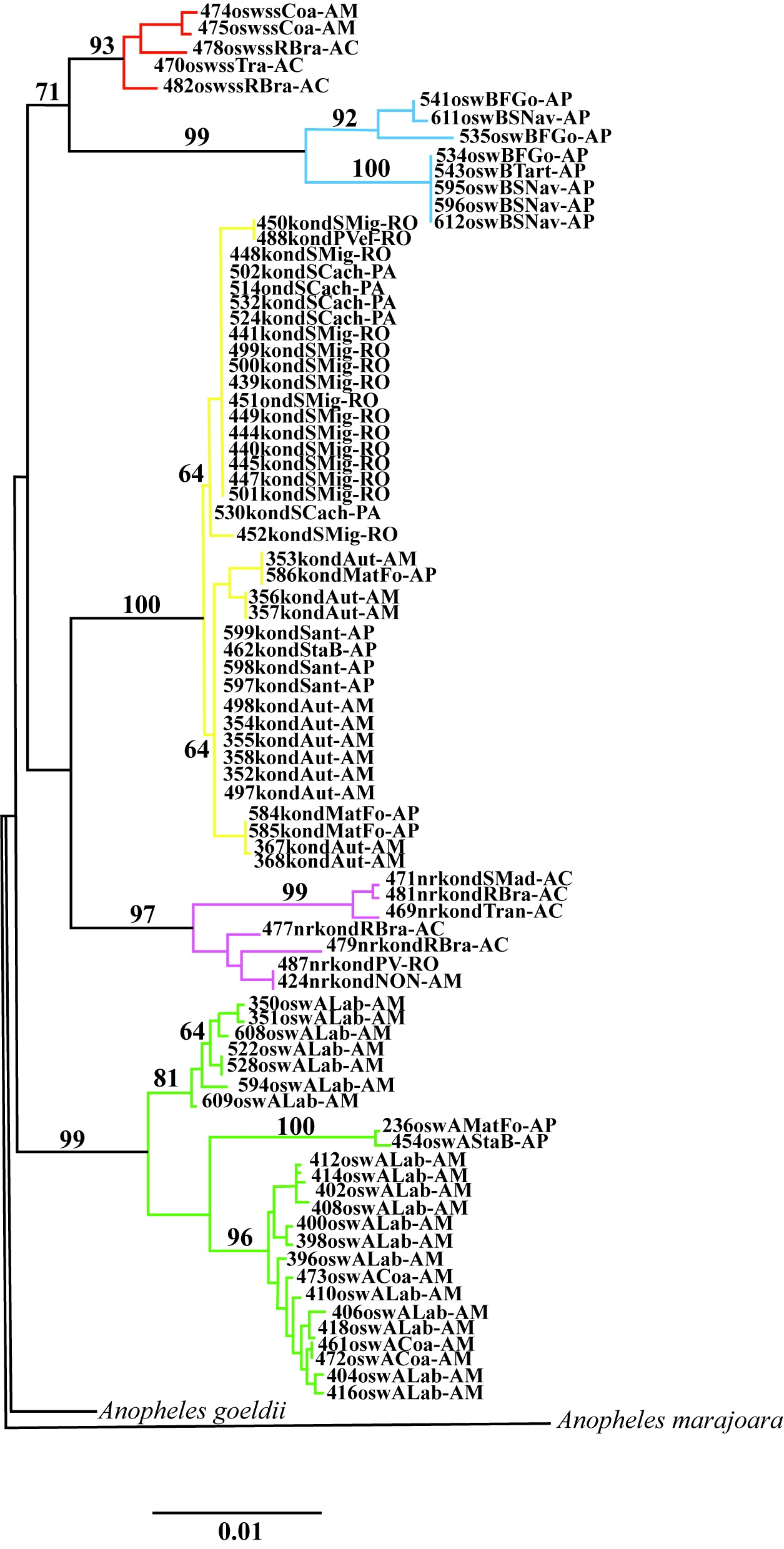

Supplement: S1 Fig — The values on the branches represent the bootstrapping support calculated with 2,000 replicates. (TIF) [file pone.0193591.s007.tif]

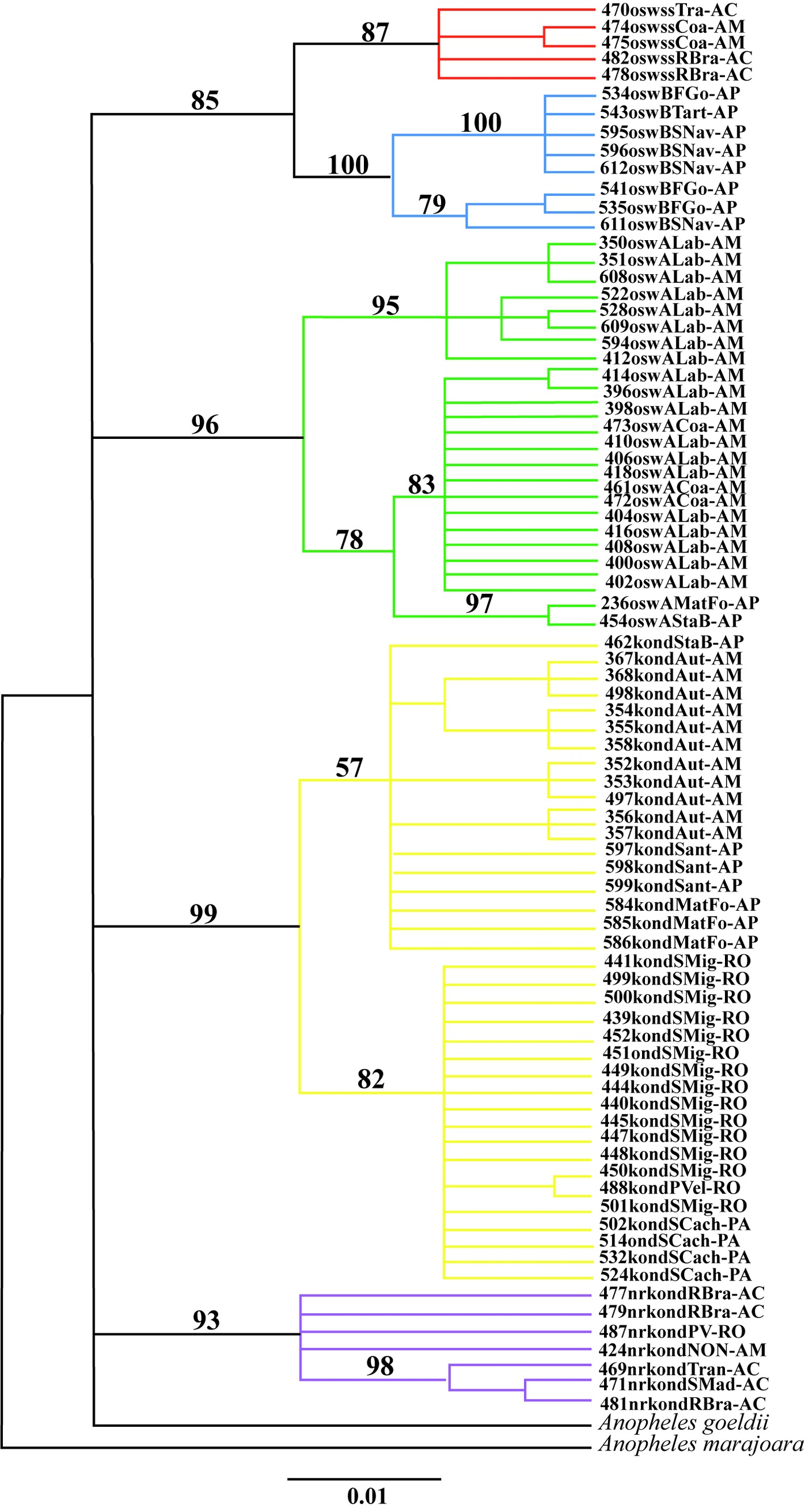

Supplement: S2 Fig — The values on the branches represent the bootstrapping support calculated with 1,000 replicates. (TIF) [file pone.0193591.s008.tif]

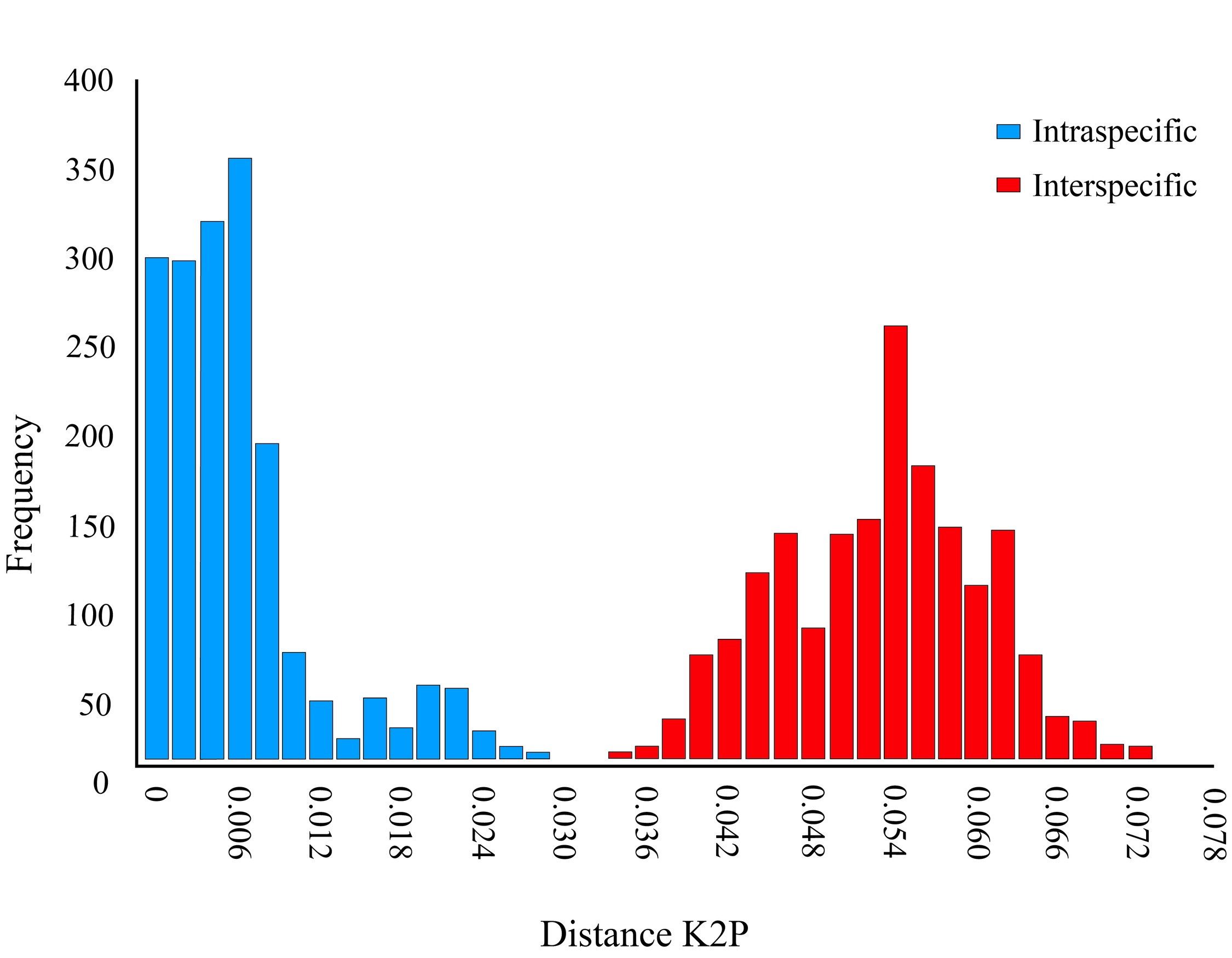

Supplement: S3 Fig — (TIF) [file pone.0193591.s009.tif]

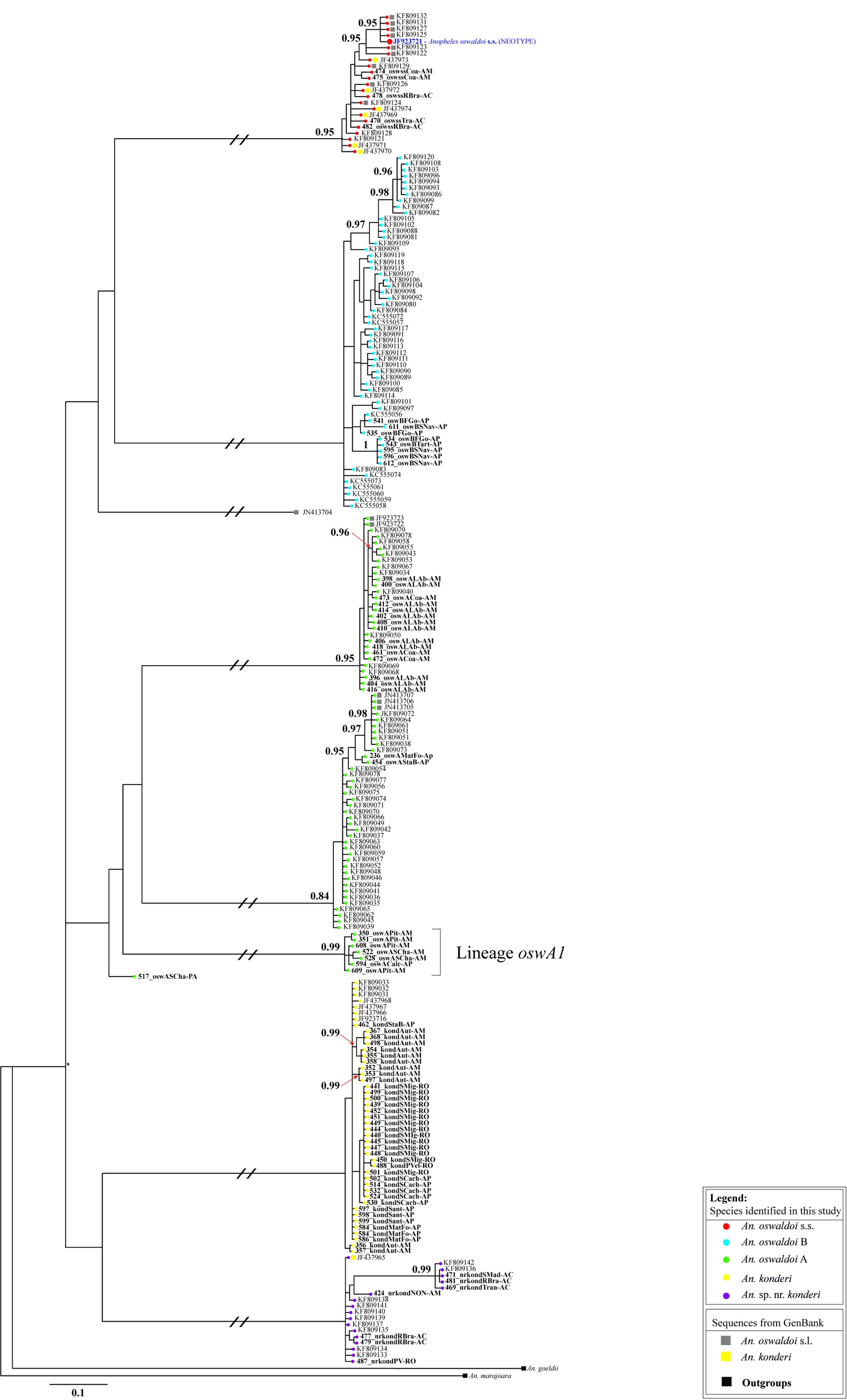

Supplement: S4 Fig — Footnote: The sequences generated in the present study are shown in bold. The sequences downloaded from GenBank are identified by the species name and access number. Red: An. oswaldoi s.s.; Blue: An. oswaldoi B; Green: An. oswaldoi A; Yellow: An. konderi; Purple: An. sp. nr. konderi. The yellow symbol (polygon) indicates the sequences of An. konderi reported by Motoki et al. [25]. (TIF) [file pone.0193591.s010.tif]
